# TCTP contains a BH3-like domain, which instead of inhibiting, activates Bcl-xL

**DOI:** 10.1038/srep19725

**Published:** 2016-01-27

**Authors:** Stéphanie Thébault, Morgane Agez, Xiaoke Chi, Johann Stojko, Vincent Cura, Stéphanie B. Telerman, Laurent Maillet, Fabien Gautier, Isabelle Billas-Massobrio, Catherine Birck, Nathalie Troffer-Charlier, Teele Karafin, Joane Honoré, Andrea Senff-Ribeiro, Sylvie Montessuit, Christopher M. Johnson, Philippe Juin, Sarah Cianférani, Jean-Claude Martinou, David W. Andrews, Robert Amson, Adam Telerman, Jean Cavarelli

**Affiliations:** 1 Département de Biologie Structurale Intégrative, Institut de Génétique et de Biologie Moléculaire et Cellulaire (IGBMC), Université de Strasbourg, CNRS UMR 7104, INSERM U964, 1 rue Laurent Fries, BP 10142, F-67404 Illkirch, France; 2 CNRS-UMR 8113, LBPA, École Normale Supérieure, 61 avenue du Président Wilson, 94235 Cachan, France; 3 Institut Gustave Roussy, Unité Inserm U981, Bâtiment B2M, 114 rue Édouard-Vaillant, 94805 Villejuif, France; 4 Sunnybrook Research Institute and Departments of Biochemistry and Medical Biophysics, University of Toronto, 2075 Bayview Ave., Toronto, Ontario, M4N 3M5, Canada; 5 Department of Chemistry and Chemical Biology, McMaster University, 1280 Main St. W. Hamilton, Ontario, L8N 3Z5, Canada; 6 Laboratoire de Spectrométrie de Masse BioOrganique (LSMBO), IPHC-DSA, Université de Strasbourg, CNRS, UMR7178, 25 rue Becquerel, 67087 Strasbourg, France; 7 King’s College London Centre for Stem Cells and Regenerative Medicine, Tower Wing, Guy’s Hospital, Great Maze Pond, London SE1 9RT, UK; 8 MRC Laboratory of Molecular Biology, Francis Crick Avenue, Cambridge Biomedical Campus, Cambridge CB2 0QH, UK; 9 Center for Cancer Research Nantes-Angers, UMR 892 Inserm - 6299 CNRS/Université de Nantes, IRS-UN, 8 Quai Moncousu - BP 70721, 44007 Nantes Cedex 1; 10 Institut de Cancérologie de l’Ouest, Centre René Gauducheau Bd Jacques Monod, 44805 Saint Herblain-Nantes cedex; 11 Department of Cell Biology, University of Geneva, 30, quai Ansermet, 1211 Geneva 4, Switzerland

## Abstract

Translationally Controlled Tumor Protein (TCTP) is anti-apoptotic, key in development and cancer, however without the typical Bcl2 family members’ structure. Here we report that TCTP contains a BH3-like domain and forms heterocomplexes with Bcl-xL. The crystal structure of a Bcl-xL deletion variant-TCTP_11–31_ complex reveals that TCTP refolds in a helical conformation upon binding the BH3-groove of Bcl-xL, although lacking the *h1*-subregion interaction. Experiments using *in vitro-vivo* reconstituted systems and TCTP^+/−^ mice indicate that TCTP activates the anti-apoptotic function of Bcl-xL, in contrast to all other BH3-proteins. Replacing the non-conserved *h1* of TCTP by that of Bax drastically increases the affinity of this hybrid for Bcl-xL, modifying its biological properties. This work reveals a novel class of BH3-proteins potentiating the anti-apoptotic function of Bcl-xL.

Members of the Bcl2 family of proteins play a critical role in governing the cell death program^1^. This family of Bcl2 proteins can be functionally divided into the pro-apoptotic and anti-apoptotic proteins. They contain subdomains that are conserved to variable extend and are called BH domains, because of their homology to Bcl2 (BH: Bcl2 homology domain). As referred to previously^2^, distant cousins of this family are termed BH3-only proteins and share the third homology domain, typically represented by Bid, Bim, Bad, Puma, Noxa and others as reviewed^1^. These BH3-only proteins are pro-apoptotic by either activating other pro-apoptotic proteins (Bax and Bak) or inhibiting the anti-apoptotic proteins (Bcl2, Bcl-xL, Mcl1, Bcl-w and A1). Hitherto, all known BH3-only proteins binding Bcl-xL inhibit its anti-apoptotic function by a well-established mechanism^3^, yet there are no BH3-proteins described activating Bcl-xL. Intriguingly the binding of these BH3-proteins to a very similar site on Bax results in the activation of its pro-apoptotic function. It remains completely unknown how the anti-apoptotic function of Bcl-xL could be potentiated.

Translationally Controlled Tumor Protein (TCTP/tpt1) is a regulator of pluripotency^4^, the cancer stem cell compartment^5^, the tumor reversion program^5^,^6^,^7^,^8^,^9^, tumor progression^5^,^6^,^7^,^8^,^9^ and certain forms of inflammatory diseases^10^. It was described as a pro-survival protein by potentiating both Mcl1^11^,^12^ and Bcl-xL^13^ and antagonizing the P53 tumor suppressor^5^. It remains unknown how TCTP regulates Bcl-xL. The initial structural analysis of TCTP indicated that it was highly conserved through phylogeny and could be related to MSS4 without any link to proteins of the Bcl2 family or any others involved in programmed cell death^14^. Given the importance in cancer of the anti-apoptotic proteins of the Bcl2 family^15^ of which Bcl-xL is a member and the focus on therapies that inhibit Bcl-xL, it becomes relevant to provide an understanding of any positive regulators of Bcl-xL.

## Results

### TCTP forms heterotetrameric complexes with Bcl-xL

By using extensive biochemical and biophysical studies (Supplementary Figs 1–6) we show that TCTP forms heterotetrameric complexes with Bcl-xL via crucial interactions between the N-Terminal region of TCTP (TCTP_11–31_) and the BH3 binding groove of Bcl-xL. Bcl-xL-Y101K mutant, which cannot bind to BH3 domains^16^, was unable to form heterocomplexes with TCTP suggesting that the interaction is with the BH3 binding pocket on Bcl-xL. ABT-737, a molecule that specifically, and with high affinity, targets the BH3-groove, abrogated formation of the TCTP/Bcl-xL complexes. There was no complex formation between Bcl-xL and full-length TCTP-R21A N-terminal mutant. These results suggest that the N-terminal part of TCTP has a BH3-like domain that interacts with the BH3-groove of Bcl-xL (Supplementary Figs 1–6).

### TCTP contains a BH3-like domain

**Figure 1:**
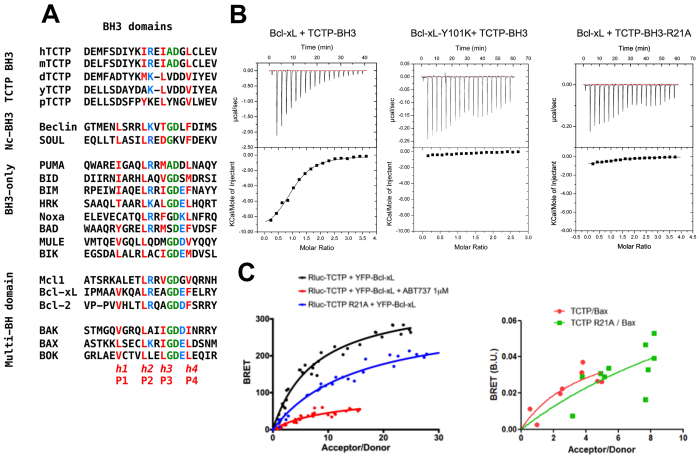
TCTP contains a non-canonical BH3 domain interacting with the BH3 groove of Bcl-xL. (**A**) Sequence alignment of the N-terminal region of TCTP (h:human; m:mouse; d:Drosophila; y:yeast; p:plant) with other BH3 domain containing proteins. In red, the conserved hydrophobic residues of the BH3 domain (named *h1* (P1), *h2* (P2), *h3* (P3) and *h4* (P4)). In green, the GD signature of BH3 domains. In blue, the conserved charged residues. (**B**) Interaction between Bcl-xL and TCTP-BH3 peptides. Calorimetric titration between (left panel) Bcl-xL (0.065 mM) and increasing amount of TCTP-BH3 (1.39 mM); (middle panel) Bcl-xL-Y101K (0.072 mM) and increasing amount of TCTP-BH3 (0.955 mM); (right panel) Bcl-xL (0.065 mM) and increasing amount of TCTP-BH3-R21A (1.26 mM). Each experiment was carried out at 30 °C in 50 mM ammonium bicarbonate pH 9. (**C**) *In cellulo* BRET analysis between TCTP and Bcl-xL, inhibition by ABT-737, lack of binding for the R21A TCTP to Bcl-xL (left panel) and Bax (right panel).

Surprisingly, aligning the amino-acid sequence of the N-terminal region of TCTP with BH3 domains from Bcl-2 family members revealed a putative BH3 domain in TCTP (Fig. 1A), suggesting that this region might bind Bcl-xL. Although the nature of the amino-acids in the *h1* (P1) sub-region of the BH3 is not conserved in TCTP, the amino-acids in *h2* (P2), *h3* (P3) and *h4* (P4), including I20, I23 and L27 are homologous to residues found in other BH3-domains that bind to the same hydrophobic groove of Bcl-xL. This putative Bcl-xL-binding domain of TCTP also contains a signature (Gly/Ala)-Asp motif between the *h3* (P3) and *h4* (P4) positions (Fig. 1A), suggesting that this region of TCTP could interact with the BH3-binding pocket of Bcl-xL.

### TCTP’s BH3 domain recognizes the BH3 groove of Bcl-xL

Isothermal Titration Calorimetry (ITC) (Fig. 1B) shows that TCTP_11–31_ peptide, containing the putative BH3-domain, binds to Bcl-xL with a dissociation constant of 12 μM. As controls for this interaction, we used two mutants: TCTP_11–31_-R21A and Bcl-xL-Y101K. TCTP_11–31_-R21A does not bind to Bcl-xL, and Bcl-xL-Y101K does not bind TCTP_11–31_ (Fig. 1B). To provide evidence that this interaction between full-length TCTP and Bcl-xL occurs at the cellular level, we measured the close proximity between transiently transfected Luciferase-fused TCTP and YFP-fused Bcl-xL by bioluminescence resonance energy transfer (BRET). As shown in Fig. 1C left panel, saturable BRET signals were observed between donor wild type TCTP and increasing levels of acceptor wild type Bcl-xL, indicative of a specific interaction between these proteins. In contrast, we detected no specific BRET signals between donor TCTP and acceptor, YFP-fused BAX, suggesting a lack of interaction between these two proteins (Fig. 1C right panel). BRET signals between TCTP and Bcl-xL were significantly inhibited by treatment of cells with the BH3 mimetic ABT-737 (Fig. 1C left panel), supporting the notion that the interaction relies on the BH3-binding groove of Bcl-xL. Further consistent with this, the R21A mutation in TCTP abrogated BRET signals between full-length TCTP and Bcl-xL (Fig. 1C left panel). Together these results suggest that BH3-TCTP binds to the BH3 groove of Bcl-xL.

### Crystal structure of Bcl-xL bound to BH3 domain of TCTP

**Figure 2:**
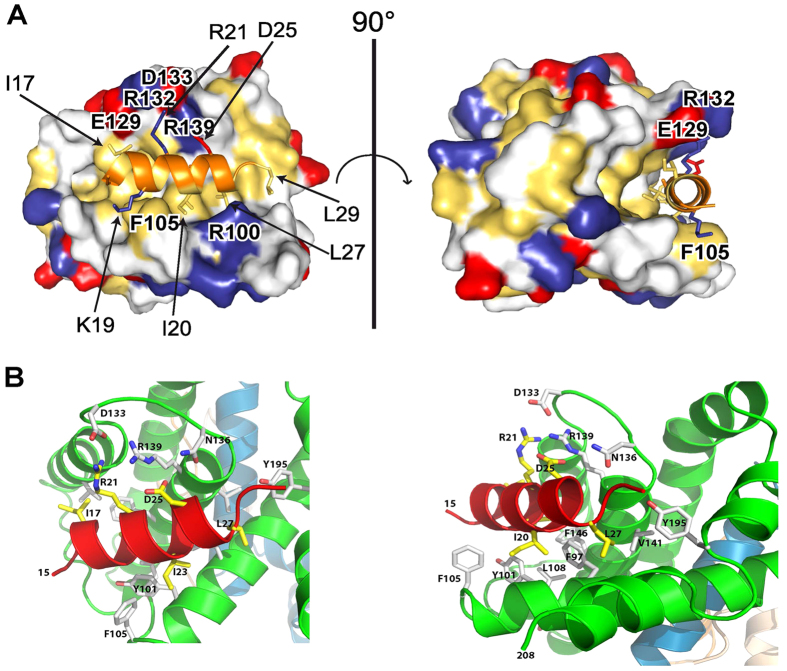
Crystal structure of Bcl-xL: TCTP-BH3 complex. (**A**) Surface diagram showing the interface between Bcl-xL and the TCTP-BH3 peptide. Bcl-xL is in the surface representation. The hydrophobic residues are drawn in yellow, positively charged residues in blue, the negatively charged residues in red, and other in white. The TCTP-BH3 peptide is represented in cartoon and colored in orange. Side chains of residues involved in the binding interface are represented as stick models. (**B**) Cartoon representation of truncated Bcl-xL bound to TCTP-BH3 domain. Two close views (left and right panel) of the interactions at the interfaces between one TCTP-BH3 peptide and the canonical BH3 groove of Bcl-xL. Residues involved in the interface are indicated and drawn stick models.

To provide structural details of the TCTP/Bcl-xL interaction, the crystal structure of a deletion variant of Bcl-xL (Bcl-xLΔ27–81ΔTM) in complex with TCTP_11–31_ has been solved and refined at 2.1 Å (Fig. 2A,B, Supplementary Fig. 7 and Table 1). This crystal structure revealed that upon binding to Bcl-xL, the residues 16–27 of TCTP_11–31_ completely refold into a 3 turn α-helix conformation that binds to the BH3-binding pocket of Bcl-xL, a hydrophobic groove created by helices α2-α5 of Bcl-xL (Fig. 2A,B). In unbound TCTP^17^, this N-terminal region stabilizes the β-sheet hydrophobic core of the entire TCTP protein (Supplementary Fig. 9). This suggests that the interaction between the full size Bcl-xL and TCTP proteins induces a substantial reorganization of the known TCTP fold.

The main difference in the binding of TCTP to the Bcl-xL groove as compared to other high affinity BH3 proteins such as Bax is the absence of the first turn of the helix, located just before the canonical *h1* (P1) motif which is non-conserved in TCTP. The functional relevance of this is addressed in the final part of this work in which this region of TCTP was replaced by the *h1* subdomain of Bax.

As observed in complexes of Bcl-xL and other BH3 peptides^18^,^19^,^20^,^21^,^22^,^23^,^24^, the interaction between Bcl-xL and the BH3 domain of TCTP is mediated by both hydrophobic and electrostatic interactions (Fig. 2A,B). The side chain of the hydrophobic residues at the *h2* (P2, I20), *h3* (P3, I23) and *h4* (P4, L27) positions form a network of van der Waals interactions with the Bcl-xL groove (Fig. 2A,B, Supplementary Figs 7,8). Three additional residues, I17, L29 and the side chain of K19 participate in this hydrophobic packing. Of note, since the canonical *h1* (P1) is missing in TCTP, I17 might counterbalance this absence by packing with the hydrophobic residues F97, Y101, F105, L108, V126, V141, F146 and Y195 of Bcl-xL in the target-binding pocket formed by helices α2-α5. Furthermore several hydrogen bonds and two salt bridge interactions contribute to the recognition process: (i) R21 of TCTP BH3 domain interacts with E129 and D133 in Bcl-xL; and (ii) D25, part of the (Gly/Ala)-Asp motif between the *h3* (P3) and *h4* (P4) positions of BH3 domain (Fig. 2A), interacts with R139 in Bcl-xL. These results explain the lack of binding of the TCTP-R21A mutant described above.

### *In vitro* and *in vivo* relevance of TCTP BH3 in regulating membrane permeabilization and the apoptotic process

**Figure 3:**
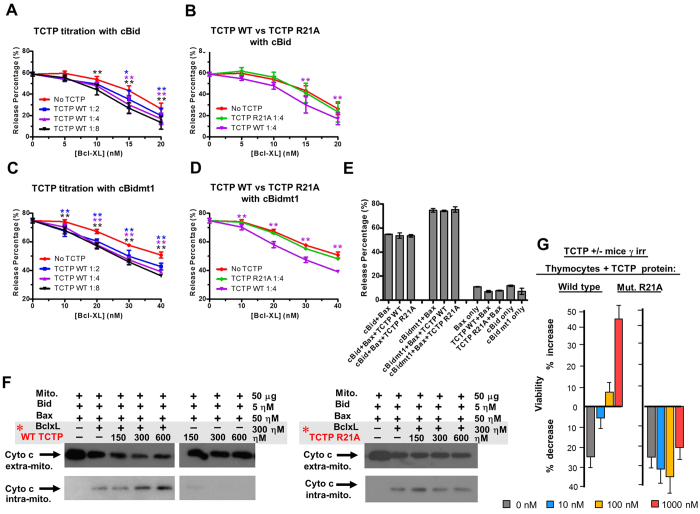
TCTP potentiates Bcl-xL inhibition of Bid/Bax. (**A**–**E**) *in vitro* reconstitution assay on liposome permeabilization. Increasing concentrations of Bcl-xL were incubated in different molecular ratios [Bcl-xL:TCTP] with TCTP WT (**A**,**C**) or with a fixed amount of TCTP R21A (**B**,**D**) at pH 9 for 45 min at 37 °C, as specified. The treated Bcl-xL was then added to reactions containing ANTS-DPX liposomes, 100 nM Bax and 20 nM of either cBid (**A**,**B**) or cBidmt1(**C**,**D**) at pH 7. Liposome permeabilization was quantified by fluorescence after 5h incubation at 37 °C where 100% release was defined as the fluorescence change due to lipid solubilization with 1% Triton X-100. Control reactions demonstrating that TCTP had no measureable effect on the function of any of the proteins other than Bcl-xL contained 320 nM pH9 treated TCTP ANTS/DPX liposomes, 100 nM Bax and 20 nM of cBid or cBidmt1 as indicated (**E**). Error bars, std. dev. n = 3. To compare between the TCTP treated groups and the Control group statistically, two-way ANOVA test was performed for (**A**–**D**) and one-way ANOVA test was performed for (**E**). Dunnett’s multiple comparisons test was used to calculate the significance of difference between the TCTP treated groups and the corresponding control group (No TCTP for (**A**–**D**), cBid+Bax or cBidmt1+Bax for **E**). Colored asterisks above each data point indicate the statistical significance (*p < 0.05; **p < 0.01, no asterisks if p ≥ 0.05). A complete statistical analysis is provided in Supplementary Table 2–4. (**F**) Permeabilization assays of mitochondrial membrane by tBid and Bax assessing cytochrome c release. Sub-optimal amounts of Bcl-xL were added as to inhibit only partially Bax mediated cytochrome *c* release. Bcl-xL and variants of TCTP were pre-incubated (TCTP ranging from 1.5–6μM and 3 μΜ Bcl-xL framed in gray with red asterisk to highlight the pre-incubation), pH 9, 30 °C then added to the mitochondria at the concentration displayed in the figure. Cyto c intra-mito: mitochondrial fraction. Cyto c extra-mito: in the supernatant, analysed by Western blot. Similar conditions were used for mutant TCTP R21A protein. (**G**) Rescue of TCTP^+/−^ haploinsufficiency: Thymocytes from TCTP^+/−^ mice were γ-irradiated (γ-irr) (2.5 Gy) and cultured with WT TCTP protein or mutant R21A TCTP at concentrations 0 to 1000 nM.

We further tackled the control that TCTP exerts on Bcl-xL in inhibiting Bax, using respectively an *in vitro* reconstituted system (Fig. 3A–E), then purified mitochondria (Fig. 3F) and thymocytes from TCTP^+/−^ haploinsufficient mice (Fig. 3G). To test the hypothesis that TCTP binding directly to Bcl-xL modifies Bcl-xL function, the impact of TCTP on membrane permeabilization was assayed using a well-established *in vitro* system in which purified recombinant proteins are used to permeabilize liposomes (Fig. 3, Supplementary Figs 10,11, Method summary and statistical analysis, Supplementary Table 2–4)^25^,^26^. This purified assay system measures direct interactions in which full-length untagged Bcl-xL inhibits permeabilization by binding full-length caspase 8 cleaved Bid (cBid) and/or full-length untagged Bax, thereby preventing Bax oligomerization in membranes^26^. To assess the effect of TCTP on Bcl-xL binding directly to Bax we used a mutant Bid (cBidmt1) that binds and activates Bax but does not bind stably to Bcl-xL^25^,^26^. In these assays liposome permeabilization by activated Bax releases the entrapped fluorophore/quencher pair 8-aminonaphthalene-1,3,6-trisulfonic acid (ANTS)/*p*-xylene-bispyridinium (DPX), resulting in a measurable increase of fluorescence. Recombinant TCTP enhanced Bcl-xL inhibition of both cBid/Bax and cBidmt1/Bax-mediated liposome permeabilization in a dose-dependent manner (Fig. 3A,C). As expected, in control experiments the TCTP R21A mutant protein had no effect on Bcl-xL activity (Fig. 3B,D). In control experiments designed to determine if TCTP affected the activity of cBid/cBidmt1-Bax induced membrane permeabilization, TCTP had no effect in reactions not containing Bcl-xL (Fig. 3E). Similar results were obtained using TCTP_11–31_ or TCTP_11–31_ R21A mutant peptides demonstrating that the putative BH3 region in TCTP is sufficient to activate Bcl-xL (Supplementary Figs 10, 11, Method summary and statistical analysis, supplementary Table 2–4). Taken together these results suggest that TCTP potentiates Bcl-xL function by increasing its interaction with Bax, and possibly also with Bid, without interfering with Bid-mediated activation of Bax.

To examine whether TCTP potentiates the anti-apoptotic effect of Bcl-xL against Bax at the mitochondrial level, we reconstituted the system with purified mitochondria^27^. When the release of cytochrome *c* is used as a probe for Bax activity on these mitochondria, we found that wild type TCTP protein (Fig. 3F) and peptide TCTP_11–31_ (Supplementary Fig. 12), in the presence of Bcl-xL, inhibit cytochrome *c* release in a concentration-dependent manner (Fig. 3F, Supplementary Fig. 12). TCTP R21A mutant protein (Fig. 3F), TCTP R21A full-length and peptide mutants (Supplementary Fig. 12) or a truncated TCTP peptide (TCTP_1–20_) (Supplementary Fig. 12), do not potentiate Bcl-xL.

TCTP deficient mice were used to investigate the biological relevance of this interaction *in vivo*. TCTP knock out mice are embryologically lethal (E 6.5–7) due to increased cell death^5^,^17^,^28^, however heterozygous animals are viable but haploinsufficient^5^. Typically, the thymocytes of these animals are more sensitive to γ-irradiation^5^. Thymocytes from irradiated TCTP^+/−^ mice were assessed for their viability in complementation assays in which either TCTP protein or peptide were added. Both, WT TCTP protein (Fig. 3G) and TCTP_1–31_ peptide (Supplementary Fig. 13), containing the BH3 domain, rescue completely the TCTP^+/−^ haploinsufficient phenotype, reducing the sensitivity to apoptosis to that found in thymocytes of wild type animals (Fig. 3G, Supplementary Fig. 13). This anti-apoptotic function of TCTP is absent in R21A mutants (Fig. 3G, Supplementary Fig. 13). The above experiments were done by taking advantage of the fact that TCTP contains in its NH_2_ terminus a protein transduction domain (PTD) with the amino-acid sequence MIIYRDLISH 29,30,31. However, all previous experiments were always performed on cell lines and not on cells directly derived from organs. It is therefore completely justified to examine how much of the full-length protein (either WT or mutant) or peptides (WT, mutant and hybrid) are penetrating efficiently irradiated thymocytes directly derived from mice, as it is the case in the present study. In all our constructs, the PTD was placed at the NH_2_ end of the peptide to be transduced in order to leave the PTD intact and the FLAG sequence was fused C-terminally. The results are shown in Supplementary Figure 14 and illustrate how efficiently TCTP full-length protein and peptides WT, mutant or hybrid are penetrating the irradiated thymocytes. The concentration used for the imaging necessitated higher amounts of peptides than the biological assays which are clearly much more sensitive. The double labeling using rabbit anti-FLAG revealed by anti-rabbit-FITC (Supplementary Fig. 14D) gives a higher background than direct labeling with anti-FLAG phyco-erythrin (Supplementary Fig. 14E-F), but both methods showed that WT or mutant TCTP peptides are equally efficiently incorporated into γ-irradiated thymocytes.

Thus in different experimental systems such as liposomes, purified mitochondria and in *ex-vivo* cell assays using TCTP^+/−^ haploinsufficient mice, binding of the BH3-region of TCTP to Bcl-xL inhibits more efficiently Bax-mediated membrane permeabilization than does Bcl-xL alone.

### Functional regulation of apoptosis by the non-canonical *h1* (P1) sub-region of TCTP’s BH3

**Figure 4:**
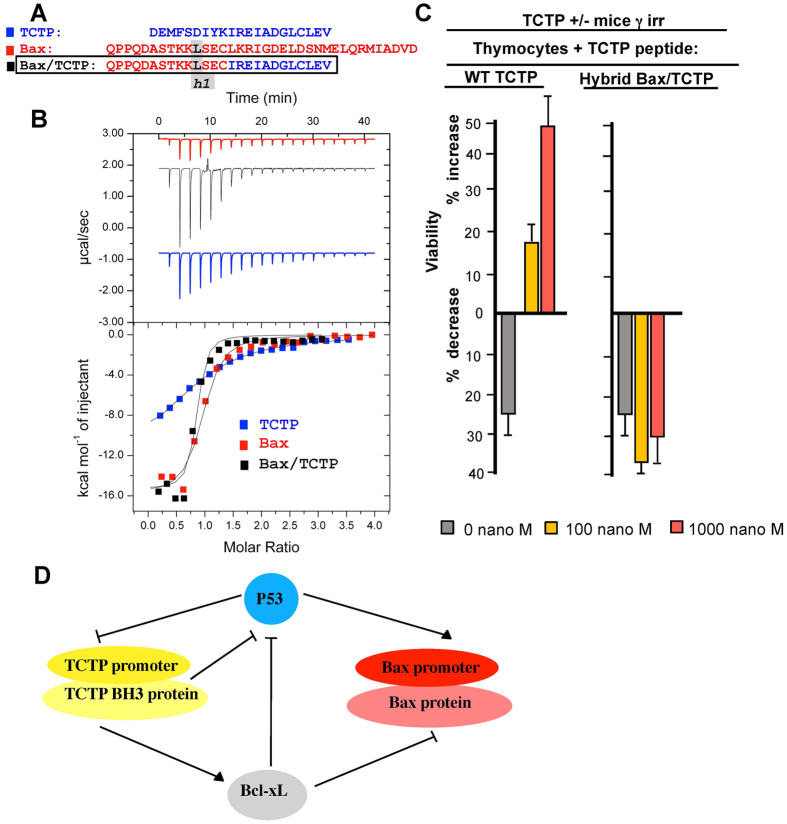
Molecular/functional analysis of the *h1* (P1) subregion of BH3-TCTP. **(A**) The three BH3 peptides (TCTP in blue, Bax in red and the hybrid Bax/TCTP aligned with the *h1* subregion framed). (**B**) Interaction between Bcl-xL and TCTP-BH3, or Bax or hybrid Bax/TCTP peptides. Calorimetric titration between Bcl-xL (0.043 mM) and increasing amount of TCTP-BH3 (0.75 mM) (blue curve); Bcl-xL (0.006 mM) and increasing amount of Bax (0.117 mM) (red curve); Bcl-xL (0.043 mM) and increasing amount of hybrid Bax/TCTP (0.65 mM) (black curve); Each experiment was carried at 30 °C in 50 mM ammonium bicarbonate pH 9. (**C**) Thymocytes from TCTP^+/−^ mice were γ-irradiated (γ-irr) (2.5 Gy) and cultured in the presence of WT TCTP_1–31_ peptide, hybrid Bax/TCTP peptide at the indicated concentrations ranging from 0 to 1000 nM. (**D**) Schematic representation of the TCTP/Bcl-xL interaction in the context of the reciprocal feedback loop between P53 and TCTP, sequestering of P53 by Bcl-xL and the control of Bax.

Based on the structural data outlined above, it appears that the *h1* (P1) sub-region in the BH3- domain of TCTP is not conserved and is not folded as in some of the other known BH3 proteins. This *h1* (P1) sub-region from different BH3 family proteins was suggested to be functionally important^32^. We therefore replaced the *h1* (P1) sub-region of BH3-TCTP by that of Bax (Fig. 4A). The Bax peptide binds with relatively high affinity resulting in a measured dissociation constant (Kd) of 200 nM to Bcl-xL, the hybrid TCTP peptide containing the Bax *h1* (P1) sub-region has a similar Kd of 500 nM, both of which are much lower than 10–15 μM Kd measured for WT TCTP (Fig. 4B).

Replacing the *h1* subdomain of TCTP by the one found in Bax completely abrogated its anti-apoptotic function when used in a complementation assay with irradiated TCTP^+/−^ haploinsufficient thymocytes, suggesting that this region confers, to a large extent, the anti-apoptotic properties of TCTP’s BH3 domain (Fig. 4C). Taken together, these results for the affinities measured by ITC for complexes between WT TCTP/Bcl-xL, Bax/Bcl-xL and the hybrid Bax(h1)-TCTP/Bcl-xL, underline the relevance of the non-canonical *h1*-subdomain in TCTP.

## Discussion

For three decades, there has been converging evidence accumulated for the role played by members of the Bcl2 family in regulating cell death during development, in physiological processes and in diseases, of which most prominently cancer^33^,^34^,^35^,^36^,^37^,^38^,^39^,^40^. However, the positive regulation and enhancement of the anti-apoptotic function of Bcl-xL remains mostly ignored^41^. In the present study, we report biochemical, structural and biological evidence that underlie this positive regulation of Bcl-xL by TCTP. TCTP is a major anti-apoptotic protein that exerts its activity on one side by direct binding to Bcl2 family members Mcl1 and Bcl-xL^11^,^12^,^13^ and on the other side by engaging into a reciprocal negative feedback loop with p53^5^. This interaction between the N-terminal region and the BH3 domain of Bcl-xL was previously reported, however it was not anticipated at that time by what mechanism this N-terminus of TCTP binds to Bcl-xL^13^. Thus, at least two major apoptotic pathways are regulated by TCTP, namely the anti-apoptotic Bcl2 family Bcl-xL and Mcl1^11^,^12^,^13^,^42^, and the pro-apoptotic p53^5^. In line with this, it was also described that reprogramming of cancer cells into revertants, which lost the malignant phenotype, parallels a drastic decrease of TCTP^6^,^7^,^8^,^9^,^43^. Also, the most aggressive forms of breast cancer have highly elevated levels of TCTP. Normal and cancer breast stem cells have an elevated level of TCTP that warrants their survival, and knocking down TCTP decreases the mammosphere-forming efficiency^5^. All these processes are closely interconnected with programmed cell death.

In this paper, we show that TCTP forms a heterotetramer with Bcl-xL and we extensively characterized these complexes using biochemical and biophysical methods. We reveal that the formation of the TCTP/Bcl-xl complex is governed by a BH3-like domain present in the N-terminal part of TCTP. The high resolution crystal structure of the purified BH3-TCTP/Bcl-xL complex highlights a substantial refolding/reorganization of the known TCTP fold and reveals at the atomic level how TCTP interacts with the BH3-binding groove of Bcl-xL. Mutational analysis of either the Bcl-xL BH3-binding groove or TCTP supports the structural data and confirms the specificity of this recognition process. Our data suggest that the interaction between the full size Bcl-xL and TCTP proteins should probably induce a substantial reorganization of the known TCTP fold. A reorganization of Bcl-xL folding is also expected, as upon binding to the TCTP BH3 peptide, helix α2 of Bcl-xL elongates and this elongation is accompanied by the flipping of F105 outwards to form a hydrophobic groove that can accommodate the binding of the BH3 peptide, while Y101 is completely buried into the core hydrophobic interface with TCTP BH3 peptide. Examples of a change of conformation of Bcl-xL induced by BH3 binding have been reported. Indeed, BH3 mimetic treatment impacts on Bcl-xL localisation^25^. Moreover, PUMA-BH3 binding to Bcl-xL partially unfolds it in such a way that its interaction with p53 via a second and distinct site is affected^23^. Whether this extends to TCTP-BH3 binding to Bcl-xL requires further investigation. In all cases these data and the present work illustrate how secondary structure refolding of proteins, as reported for the widely-known examples of prion proteins^44^ and few others examples, may be a key event in biology, modulating the conformation of proteins in the cell and generating different active and functional states^45^,^46^.

This regulation of the anti-apoptotic process by the BH3 domain of TCTP was further corroborated in *in vitro* reconstituted systems, where the sole presence of Bax, Bid or Bim, Bcl-xL and TCTP is clearly sufficient to control membrane permeabilization. This was further confirmed on purified mitochondria and for both of these systems a mutation is the BH3 domain of TCTP abrogated its function. This was ultimately evidenced on TCTP heterozygous haploinsufficient mice in which after γ-irradiation, the apoptosis process is rescued by wild type TCTP protein or peptide but not anymore by the mutant. We also tried to understand where resides the major difference between the binding of a negative pro-apoptotic regulator such as Bax and a positive regulator such as TCTP with regard to their respective interaction to Bcl-xL. Bax binds with a much higher affinity to Bcl-xL than TCTP does, and a hybrid mutant where the h1 subregion of TCTP was replaced by this domain from Bax results in a total loss of the positive regulation of Bcl-xL by TCTP. This hybrid mutant was not able anymore to rescue the haploinsufficient phenotype TCTP^+/−^ murine thymocytes following γ-irradiation.

Our data highlight how subtle changes between two BH3 domains, more specifically here in the *h1*-subdomain, resulting in only a slight difference in the mode of recognition with the BH3 groove of Bcl-xL, reveal a significant increase in binding affinity, hence completely modifying the biological outcome. Combined with the structural analysis (Fig. 2A,B) where this region folds differently into Bcl-xL, these results might suggest a model wherein a rapid exchange of proteins bound to Bcl-xL occurs in favor of Bax/Bcl-xL complexes and, as such, TCTP could prime and activate Bcl-xL against Bax. It should be borne in mind that in exponentially growing cells or cancer cells where apoptosis is inhibited, TCTP levels are extremely high, even above actin^47^, and that in such conditions low affinity of TCTP/Bcl-xL would be compensated by abundance.

Altogether our results demonstrate that TCTP contains a non-canonical but functional BH3-domain that binds to -and unexpectedly activates- the anti-apoptotic function of Bcl-xL. We speculate that activation is achieved by virtue of the low affinity of the interaction as mutations that increase the affinity of the interaction abolish the activity of TCTP. Perhaps, the BH3-TCTP competes with Bcl-xL H9 for the BH3-binding pocket on Bcl-xL, partially activating Bcl-xL. It would not be surprising if other proteins with non-canonical BH3 peptides potentiating the anti-apoptotic effect of specific Bcl2 family members were identified. That BH3-proteins can either increase or decrease the activity of Bcl-xL increases the complexity of the system but also highlights the potential for identification of new therapeutic targets which would not only “hit” the anti-apoptotic proteins but also positive BH3-like regulators. Our demonstration of the interplay between TCTP and Bcl-xL might also shed a new light on the effects of TCTP on p53, of which Bcl-xL is a binding partner^5^,^48^. This might connect Bcl-xL and p53 to the regulation of other processes regulated by TCTP, such as cell shape and migration. Of note, it is known that TCTP interacts with the cytoskeleton^49^, more specifically with centrosomes and indirectly with microtubules^50^. Moreover, the inverse correlation between TCTP/RhoA and p53/cyclin A/actin expression suggests a common regulation for those proteins and their pathways^51^. TCTP is also implicated in the epithelial to mesenchymal transition^31^. From a more comprehensive perspective, accumulating knowledge suggest that TCTP functions as a “smart linker” unifying, regulating and ultimately defining the outcome of a wide variety of biological processes ranging from pluripotency to cell shape, cell death, tumorigenicity and tumor reversion. It exerts its function on a range of target-proteins and the data presented here indicate that both TCTP and the target -at least for TCTP/Bcl-xL- undergo major structural modifications which are probably at the basis of such a widespread biological effect of TCTP.

## Methods

### Constructs expression and mutagenesis

For expression as a GST-fusion protein, TCTP was cloned into a pHGGWA plasmid^52^ using the GatewayTM Technology (Invitrogen). The TCTP gene was amplified by PCR and cloned into a pDONR207 plasmid by BP reaction and then subcloned into pHGGWA plasmid by the LR reaction as described in Busso *et al.*^52^. See Supplementary Information for full details.

#### Bioluminescence resonance energy transfer (BRET)

BRET assays were performed as indicated in Bah *et al.*^41^. See Supplementary Information for full details.

#### Protein expression and purification

See Supplementary Information for full details.

#### Peptides used for *in vitro* assays and *in vivo* assays

See Supplementary Information for full details.

### Complementation experiments

In order to complement the haploinsufficient TCTP^+/−^ mice with exogenous TCTP, we took advantage of the fact that the first N-terminal fragment of TCTP functions as a Protein Transduction Domain (PTD), enabling TCTP to penetrate into the cell (Supplementary Figs 13–14D) by a mechanism hitherto not well understood^30^,^53^,^54^,^55^. Thymocytes from wild type or TCTP^+/−^ mice were irradiated (2.5 Gy of γ-irradiation) and cultured overnight in the presence of TCTP protein (Wild type or mutant TCTP protein bearing the mutation R21A) or TCTP peptide (wild type TCTP peptide 1–31, mutant TCTP peptide bearing the mutation R21A, or peptide 1–20) at concentrations ranging from 0–1000 nM.

### Isothermal Titration Calorimetry experiments

Titrations were done by isothermal titration calorimetry (ITC) assays using an ITC200 calorimeter from GE-Microcal. See Supplementary Information for full details.

### Crystallization, X-ray data collection, structure determination, and refinement

High-throughput crystallization screening was performed using a Mosquito liquid transfer robot (TTP Labtech) and the sitting-drop vapor diffusion method. Crystals were obtained at 20 °C by mixing 0.2 μL of reservoir solution (100 mM Pipes pH 7, 1.5M Tris-sodium citrate) with 0.2 μL of 300 μM of Bcl-xL (Δ27–81ΔCT) + 1 mM TCTP_11–31_ solution in buffer containing 50 mM ammonium bicarbonate pH 9.3 and by equilibrating the mixture against 40 μl of reservoir solution. A 2.1 Å resolution data set was collected at 100 K on beamline ID29 at the European Synchrotron Radiation Facility with a Pilatus 6MF detector. The crystal belongs to space group P4_1_2_1_2 with unit cell dimensions a = 100.36 Å, b = 100.36 Å, c = 105.04Å and α = β = γ = 90°. Diffraction data were processed, integrated, and scaled with XDS^56^ and HKL2000^57^. The structure of human Bcl-xL/TCTP_11–31_ complex was solved by molecular replacement using the program Phenix^58^ and coordinates from the human Bcl-xL-Beclin complex (Protein Data Bank code 2P1L^20^) as a search model. The model was built with Coot^59^ and refined with Phenix^58^ and Buster^60^. The statistics are summarized in Table 1. See Supplementary Information for full details. Coordinates of the refined structural model and structure factors have been deposited to the Protein Data Bank (PDB) with the accession code 4Z9V.

#### Interaction experiments

When not specified, proteins tested for binding experiments were mixed at 100 μM, dialyzed against 10 mM CHES pH 9, 50 mM NaCl, 1 mM DTT or TCEP, depending on the analysis, and heated overnight at 30 °C.

#### Gel filtration

For binding experiments, 100 to 200PL of protein mixture were loaded on a size exclusion chromatography (S200, GE Healthcare) column, while elution profiles were measured by UV absorption at 280 nm.

### Size-Exclusion Chromatography coupled to Multi-Angle Light Scattering (SEC-MALS)

SEC-MALS experiments were performed on a multi-angle light scattering detector (miniDAWN TREOS, Wyatt Technologies) coupled in-line with SEC and an interferometric refractometer (Optilab T-rEX, Wyatt Technologies). See Supplementary Information for full details.

#### Native mass spectrometry

Prior to any mass spectrometry experiment, protein buffer was exchanged twice against a 50 mM ammonium acetate (NH4Ac) solution at pH 9.0 using microcentrifuge gel filtration columns (Zeba 0.5 ml, Thermo Scientific, Rockford, IL). Protein concentration was determined spectrophotometrically. NanoESI-MS measurements were carried out on an electrospray quadrupole-time-of-flight mass spectrometer (Synapt G2 HDMS, Waters, Manchester, UK) equipped with an automated chip-based nanoESI source (Triversa Nanomate, Advion Biosciences, Ithaca, NY) operating in the positive ion mode. See Supplementary Information for full details.

#### *in vitro* system

purified recombinant proteins are used to permeabilize liposomes as described before^25^,^26^. See Supplementary Information for full details.

#### Cytochrome *c* release assay

Mitochondria from mouse liver were purified as described by Eskes *et al.*^61^. See Supplementary Information for full details.

All experiments were performed in accordance with relevant guidelines and regulation. All experimental protocols described in this paper were approved by CNRS and INSERM. All animal studies were carried out in the restricted facilities provided by the CNRS, INSERM and Institut Gustave Roussy, by authorized personnel following the prescribed rules.

## Additional Information

**Accession numbers**: Coordinates of the refined structural model and structure factors have been deposited to the Protein Data Bank (PDB) with the accession code 4Z9V.

**How to cite this article**: Thébault, S. *et al.* TCTP contains a BH3-like domain, which instead of inhibiting, activates Bcl-xL. *Sci. Rep.* **6**, 19725; doi: 10.1038/srep19725 (2016).

## Supplementary Material

Supplementary Information

## Figures and Tables

**Table 1:** X-ray Crystallographic Data collection and structure refinement statistics.

Data collection	
X-ray source	ESRF ID29
Wavelength (Å)	0.97625
Data collection temperature (K)	100
Detector	Pilatus 6MF
Crystal-detector distance (mm)	383.18
Total rotation range (°)	180
Exposure range (°) and time (s) per image	0.1, 0.04
Mosaicity (°)	0.474
Cell parameters (Å)	a = 100.36, b = 100.36, c = 105.04, α = β = γ = 90°
Space group	P4_1_2_1_2
Resolution range (outer shell) (Å)	46.50-2.10 (2.23–2.10)
Total number of reflections	404573 (61254)
Number of unique reflections	31973 (4976)
Completeness (%)	99.7 (98.4)
Multiplicity	12.7 (12.2)
<I/σ(I)>	19.2 (1.1)
Rmerge	0.074 (2.2)
*R*_meas_	0.077 (2.320)
CC_1/2_	0.999 (0.436)
Refinement	
Resolution range (Å)	37.45 − 2.10 (2.174 − 2.10)
R-work/R-free	0.185 (0.347)/0.221 (0.361)
Number of atoms	3444
Protein; ligands; water	3377; 10; 57
Protein residues	418
RMS deviations from ideal bond lengths (Å)*	0.003
RMS deviations from ideal bond angles (°)*	0.77
Ramachandran favored (%)	98
Ramachandran outliers (%)	0
Average B-factor (Å^2^)	69.10
macromolecules; ligands; solvent	69.2; 88.10; 59.10
Molprobity Validation	
Rotamer and C-beta outliers	0.3%; 0.
Clashscore and Overall score	2.42; 1.01

^†^mean *I*/σ(*I*) falls below 2.0 in the outer shell at 2.2 Å. R_meas_ is the redundancy-independent merging *R* factor. Highest resolution shell is shown in parenthesis.
