# Supplementary material for: TCTP contains a BH3-like domain, which instead of inhibiting, activates Bcl-xL
Source: Sci Rep. 2016 Jan 27;6:19725. doi: 10.1038/srep19725 (PMC4728560; doi:10.1038/srep19725)
Supplement: Supplementary Information [file srep19725-s1.pdf]

Supplementary Information

TCTP contains a BH3-like domain, which instead of inhibiting, activates Bcl-xL.

Stéphanie Thébault, Morgane Agez, Xiaoke Chi, Johann Stojko, Vincent Cura, Stéphanie B. Telerman, Laurent Maillet, Fabien Gautier, Isabelle Billas-Massobrio, Catherine Birck, Nathalie Troffer-Charlier, Teele Karafin, Joane Honoré, Andrea Senff-Ribeiro, Sylvie Montessuit Christopher M. Johnson, Philippe Juin, Sarah Cianférani, Jean-Claude Martinou, David W. Andrews, Robert Amson, Adam Telerman* and Jean Cavarelli*

*Corresponding authors:
Adam Telerman (atelerman@gmail.com) and Jean Cavarelli (cava@igbmc.fr)

Table of Content:

Supplementary Text
Supplementary Figure 1 to 14
Supplementary Table 1-4
Supplementary Methods
Supplementary References

TCTP preferentially recognizes dimers of Bcl-xL

Bcl-xL wild type (Bcl-xL WT) is a 233 amino-acid protein consisting of 9 helices (D1-D9), represented in cylinders, an Unfolded Domain (UD) and a Terminus Membrane Domain (TMD) formed by helix α9 (**Supplementary Figure 1**). In this study, we used three constructs of Bcl-xL which lacked the C-terminal α9 transmembrane helix (ΔCT) and different lengths of the loop between helices α1 and α2: Bcl-xL(ΔCT), Bcl-xL(Δ45-84ΔCT)(Bcl-xL lacking amino-acids 45-84) and Bcl-xL(Δ27-81ΔCT) (Bcl-xL lacking amino-acids 27-81). Nevertheless, all these 3 constructs conserve their BH3 binding properties. The structure of human TCTP[1] contains three α-helices (α1, α2, α3) and nine β-strands arranged in two distorted β-sheets, forming two hydrophobic cores. A α-helical hairpin is formed by the two long helices α2 and α3 (colored in yellow) and protects one face of the central β-sheet (colored in green and red).

The first construct, Bcl-xL(ΔCT), was incubated with full-length TCTP in a 1:1 ratio (each protein at 100μM) at 4°C and 30°C. Complex formation was then assessed by size exclusion chromatography (SEC). **Supplementary Figure 2A** and **2B** display the elution chromatogram of purified proteins, TCTP and Bcl-xL(ΔCT), and the mixture TCTP+Bcl-xL(ΔCT), respectively. As observed in **Supplementary Figure 2A**, Bcl-xL(ΔCT) presents a mixture of monomers and dimers as already described in the literature[2-4].

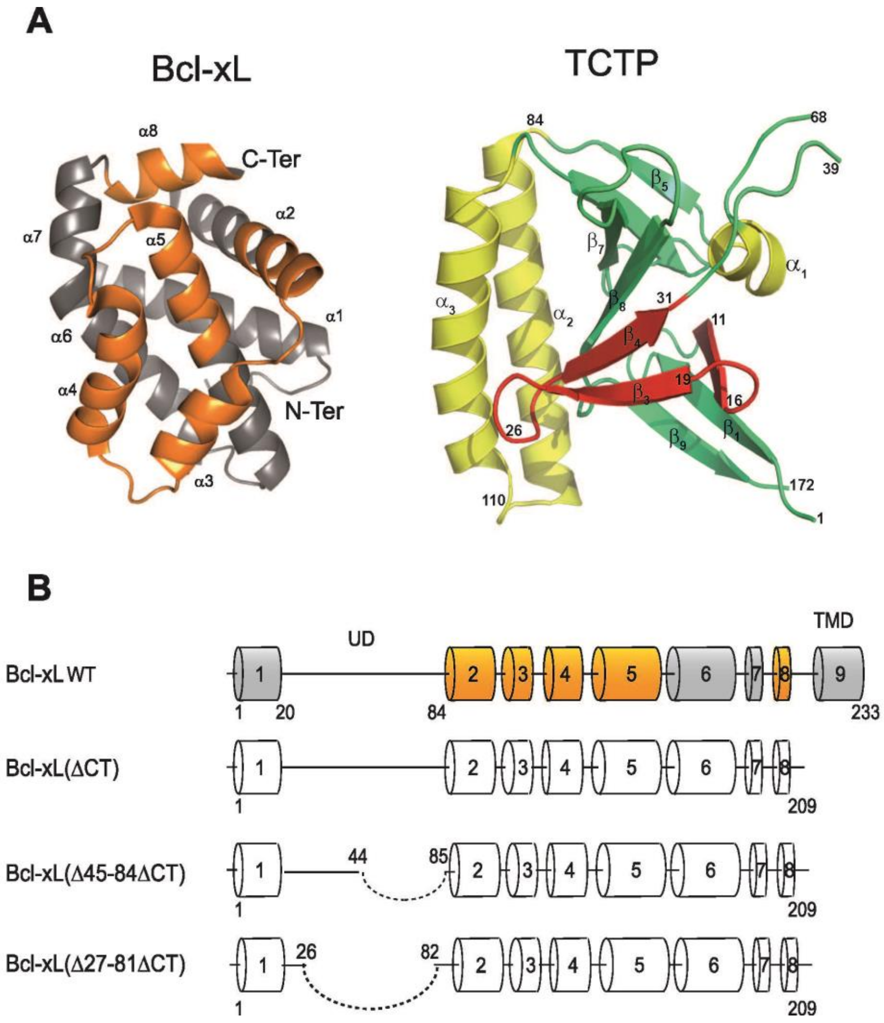

Supplementary Figure 1: Constructions of TCTP and Bcl-xL

A) Cartoon representation of Bcl-xL (Left panel, PDB entry 1R2D) and TCTP (Right panel, PDB entry 1YZ1). The BH3 groove of BCL-xL binding to BH3-only Bcl-2 proteins, colored in orange, contains the C-terminal part of α2, helices α3-D α4, the N-terminal part of α5 and the helix α8. In the structure of human TCTP α-helical hairpin is formed by the two long helices α2 and α3 (colored in yellow) and protects one face of the central β-sheet (colored in green and red). Drawings were produced with Pymol[5]. **B)** Schematic representation of Bcl-xL constructs used in this study (UD; Unfolded Domain; TMD; Trans-Membrane Domain; helices are numbered from 1 to 9).

Supplementary Figure 2 shows that the elution profile of the mixture of (TCTP+Bcl-xL(ΔCT)) incubated at 4°C corresponds to that of isolated proteins, which suggests that at low temperature no complex is formed. On the contrary, at 30°C, formation of a TCTP:Bcl-xL(ΔCT) complex is easily detectable as shown by the appearance of a new peak eluted at a significantly smaller elution volume than for both proteins separately. SDS-PAGE analysis of the corresponding fractions also shows that this new peak contains both proteins and therefore validates the formation of a complex between TCTP and Bcl-xL(ΔCT). However, only a small proportion of TCTP and Bcl-xL(ΔCT) seems to be involved in the complex. Thus, we tested the effect of concentration and protein ratio on TCTP:Bcl-xL complex formation. **Supplementary Figure 2C** shows the elution profile of TCTP + Bcl-xL(ΔCT) incubated at higher protein concentrations (each protein at 350μM) at 30°C in equimolar conditions. As expected, the elution peak corresponding to TCTP:Bcl-xL(ΔCT) complex appears relatively more intense. Nevertheless, a significant proportion of free TCTP and Bcl-xL(ΔCT) remains unbound, as confirmed by SDS-PAGE. **Supplementary Figure 2D** displays the elution profile of TCTP+Bcl-xL(ΔCT) incubated at 30°C in a 1:3 molar ratio (150μM TCTP:450μM Bcl-xL(ΔCT)). In those conditions, only a small amount of TCTP is still unbound compared to previous experiments. Thus, an excess of Bcl-xL(ΔCT) will displace the binding equilibrium towards TCTP:Bcl-xL(ΔCT) complex formation. Interestingly, for each condition, signal intensity of the elution peak corresponding to the dimer of Bcl-xL(ΔCT) decreases significantly more than the one corresponding to the monomer. These experiments suggest that TCTP preferentially recognizes dimeric forms of Bcl-xL(ΔCT) and that TCTP:Bcl-xL interaction is promoted by heating at 30°C.

The second construct, Bcl-xL(Δ45-84ΔCT), lacks a short portion of the α1- α2 loop and can also

be purified as a mixture of monomers and dimers. We therefore tested if TCTP binding could discriminate between dimers and monomers of Bcl-xL(Δ45-84ΔCT). Taking advantage of previous results, we incubated TCTP with 3 molar excess of Bcl-xL(Δ45-84ΔCT) (150μM TCTP:450PM Bcl-xL(ΔCT)) at 30°C (**Supplementary Figure 2E**). The elution profile confirms the formation of a TCTP:Bcl-xL(Δ45-84ΔCT) complex, while SDS-PAGE analysis shows that TCTP is predominantly bound to Bcl-xL(Δ45-84ΔCT). As for Bcl-xL(ΔCT), dimeric forms of Bcl-xL(Δ45-84ΔCT) seem to be preferentially targeted by TCTP.

The third construct of Bcl-xL, Bcl-xL(Δ27-81ΔCT), presents a shorter loop between helices α1 and α2 than Bcl-xL(Δ45-84ΔCT) and is mainly purified as dimers[4] (**Supplementary Figure 2F**). To verify the hypothesis by which TCTP would preferentially bind the dimeric form of Bcl-xL, we incubated TCTP and Bcl-xL(Δ27-81ΔCT) at 30°C in an equimolar ratio (each protein at 100μM) before SEC characterization. As depicted, results clearly show the formation of a hetero-complex between TCTP and Bcl-xL(Δ27-ΔCT), corroborated by SDS-PAGE analyses. However, the elution profile of the TCTP:Bcl-xL(Δ27-ΔCT) complex is broad and asymmetrical, reflecting a heterogeneous composition. Conversely, TCTP:Bcl-xL(Δ45-84ΔCT) and TCTP:Bcl-xL(ΔCT) complexes require an excess of Bcl-xL but form a more homogeneous population, as observed by a more symmetrical elution peak. Otherwise, while most of dimeric forms of Bcl-xL(ΔCT) and Bcl-xL(Δ45-84ΔCT) disappear after incubation with TCTP, the absorbance of the monomeric form barely decreases. All these experiments suggest that TCTP preferentially binds the dimeric form of Bcl-xL. This also indicates that the TCTP:Bcl-xL interaction is promoted by heating at 30°C, and that the loop between α1 and α2 (corresponding to the residues 27 to 81 of Bcl-xL is not necessary for its binding to TCTP.

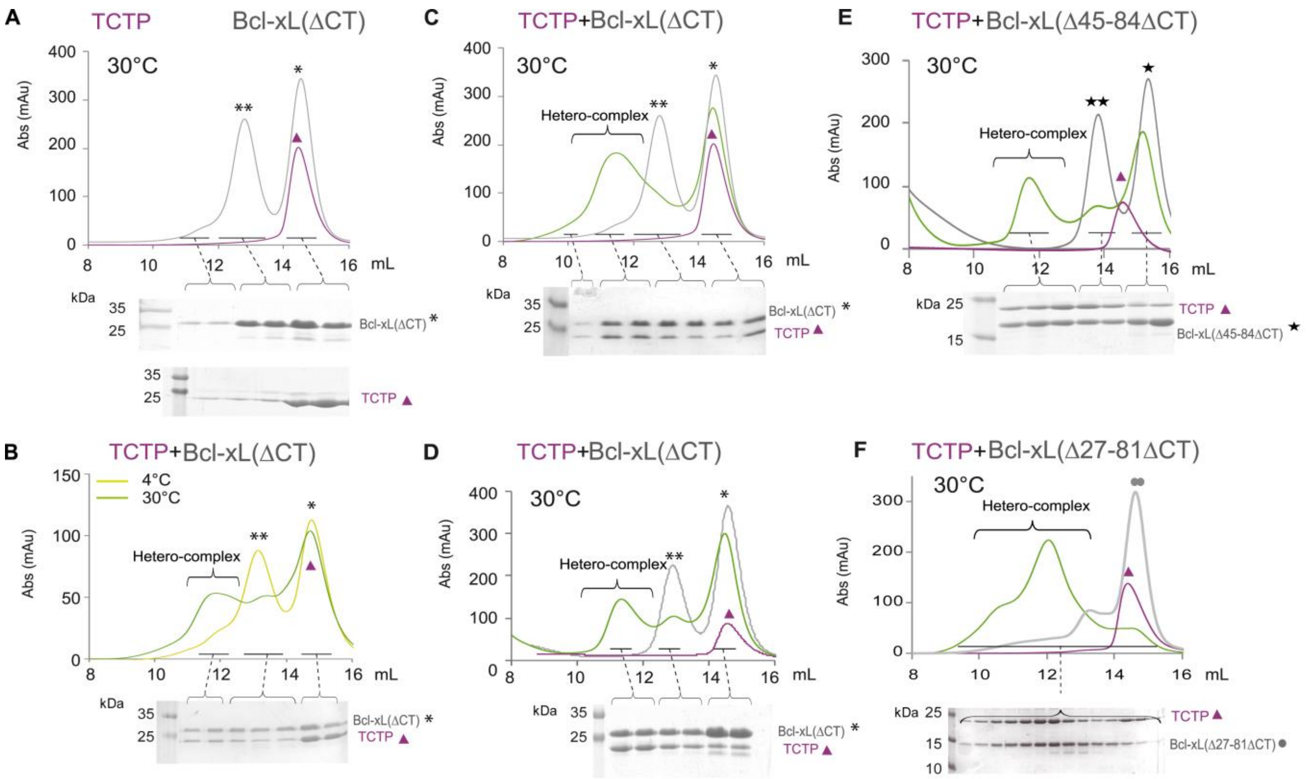

Supplementary Figure 2: Co-purification of TCTP:Bcl-xL complexes

A) TCTP (magenta) and Bcl-xL(ΔCT) (grey). TCTP elutes as a monomer (▲) at 14.5mL, Bcl-xL(ΔCT) elutes as a mixture of dimers (**) at 12.9mL and monomers (*) at 14.7mL. Corresponding 12% SDS-PAGE analyses are depicted bellow. Isolation of a complex between TCTP and Bcl-xL(ΔCT). **B**) Superimposition of analytical gel filtration profiles of TCTP + Bcl-xL(ΔCT) with 100µM TCTP and 100µM Bcl-xL(ΔCT) at 4°C (light green) and 30°C (dark green) ; **C**) Superimposition of analytical gel filtration profiles of TCTP (magenta), Bcl-xL(ΔCT)(grey) and TCTP + Bcl-xL(ΔCT) with 350µM TCTP and Bcl-xL(ΔCT) at 30°C (green); **D)** Superimposition of analytical gel filtration profiles of TCTP (magenta), Bcl-xL(ΔCT) (grey) and TCTP + Bcl-xL(ΔCT) with 150µM TCTP and 400µM Bcl-xL(ΔCT) at 30°C(green). Isolation of a complex between TCTP and Bcl-xL(Δ45-84-ΔCT): **E**) Analytical gel filtration profiles of TCTP (magenta), Bcl-xL(Δ45-84-ΔCT) (grey) and TCTP + Bcl-xL(45-84-ΔCT) (green) at 30°C with 150µM TCTP and 400µM Bcl-xL(Δ45-84-ΔCT; Bcl-xL(Δ45-84-ΔCT) elutes as a mixture of dimers (**) at 13.8mL and monomers (*) at 15.4mL. Isolation of a

complex between TCTP and Bcl-xL (Δ27-81-ΔCT): **F**) Analytical gel filtration profiles of TCTP (magenta), Bcl-xL(Δ27-81ΔCT)(grey) and TCTP + Bcl-xL(Δ27-81ΔCT) with 100µM TCTP and Bcl-xL(Δ27-81ΔCT) at 30°C (green) , Bcl-xLΔ27-81-ΔCT elutes as a dimer (●●) at 14.8mL.

Now that we could isolate a TCTP:Bcl-xL(Δ27-81ΔCT) complex, we decided to further characterize its pH and ionic strength dependency. First, complex formation was monitored at different pHs (from 7.0 to 9.0) by size exclusion chromatography (**Supplementary Figure 3A**). The elution peak corresponding to the TCTP:Bcl-xL(Δ27-81ΔCT) complex decreases between pH 9.0 and pH 8.0 while the one corresponding to the free proteins increases. Almost no complex is obtained at pH 7.0. These results suggest that the TCTP:Bcl-xL complex is highly sensitive to pH and stabilized at alkaline pH. We then tested the formation of this complex with increasing ionic strengths (50mM and 200mM NaCl, **Supplementary Figure 3B**). At 200mM, the elution peak corresponding to the complex decreases drastically. Those results suggest that high salt concentrations destabilize the formation of TCTP:Bcl-xL interface.

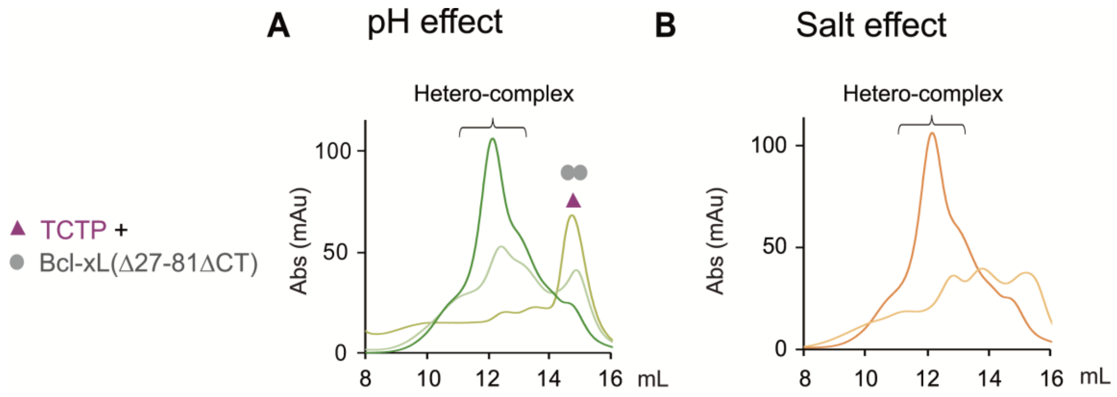

Supplementary Figure 3. Biochemical characteristics of the TCTP: Bcl-xL(Δ27-81-ΔCT)

complex . A) pH effect on TCTP:Bcl-xL(Δ27-81ΔCT) complex: analytical gelfiltration profiles of TCTP+Bcl-xL(Δ27-81ΔCT) at pH 9 (dark green), pH 8 (green) and pH 7 (light green). **B**) Salt effect on TCTP:Bcl-xL(Δ27-81ΔCT) complex: analytical gel filtration profiles of TCTP + Bcl-xL(Δ27-81ΔCT) at 50mM NaCl (orange) and 200mMNaCl (light orange).

TCTP forms hetero-tetrameric complexes with Bcl-xL

We performed Size Exclusion Chromatography coupled to Multi-Angle Laser Light Spectrometry (SEC-MALS), and native mass spectrometry (MS) on TCTP:Bcl-xL complexes to determine their binding stoichiometries. The SEC-MALS method provides information on sample purity and monodispersity (SEC) and a direct measure of the particle's molecular weight (MALS). The SEC-MALS profile of TCTP+Bcl-xL(Δ27-81ΔCT) incubated in an equimolar ratio at 30°C shows four populations (**Supplementary Figure 4A**). The two peaks at higher elution volume correspond to Bcl-xL(Δ27-81ΔCT) homo-dimers (with an apparent molecular mass of 36.1 kDa ± 0.4% at 13.8 mL) and TCTP monomers (with an apparent molecular mass of 20.9 kDa ± 2.9% at 14.1 mL), as confirmed by SEC-MALS analyses of individual proteins (**Supplementary Figure 5**). These results are in agreement with the calculated molecular masses of 35.54 kDa and 19.65 kDa for Bcl-xL(Δ27-81ΔCT) homodimers and TCTP monomers, respectively. The first eluted peak, displaying an elution volume of 11.5 mL, is associated with an apparent mass of 73.5 kDa ± 0.1%, which is consistent with a hetero-tetrameric complex containing 2*TCTP+2*Bcl-xL(Δ27-81ΔCT) molecules (calculated molecular mass of 74.8 kDa). The second eluted peak (annotated B), which has an apparent molecular mass of 45.9 kDa ± 0.1%, might be a dimer of TCTP. Indeed, the 2*TCTP species has a calculated mass of 39.3kDa, but the observed higher mass could be due to the close elution of the hetero complex 2*TCTP+2*Bcl-xL(Δ27-81ΔCT).

To further determine TCTP:Bcl-xL binding stoichiometries, we performed native ESI-MS analysis, a powerful technique to study non-covalent interactions allying sensitivity to high resolution detection. After confirming the exact entire mass of individual partners in denaturing conditions (**Supplementary Table 1**), native MS experiments were performed at 30°C in equimolar protein ratios (each protein at 100µM).

Supplementary Figure 4B shows native mass spectra obtained for TCTP (upper panel), Bcl-xL(Δ27-81ΔCT) (middle panel) and the TCTP+Bcl-xL(Δ27-81ΔCT) complex (lower panel) after dilution to 5µM. TCTP is mainly detected as a monomer (>90%) with a molecular mass of 19651 ± 1 Da while Bcl-xL(Δ27-81ΔCT) is predominantly observed in its dimeric form (35542 ± 1 Da), with a small portion of monomeric forms (17771 ± 1 Da). Measured molecular masses are in good agreement with the calculated ones (**Supplementary Table 1**). For the TCTP+Bcl-xLΔ27-81ΔCT sample, the most intense ion series at high m/z corresponds to the formation of a hetero-tetrameric species constituted of 2*TCTP+2*Bcl-xL(Δ27-81ΔCT) with a measured mass of 74852 ± 4 Da (calculated mass: 74844 Da). Altogether, native ESI-MS and SEC-MALS results consistently suggest the presence of a heterotetrameric complex composed of two molecules of TCTP and two molecules of Bcl-xL(Δ27-81ΔCT).

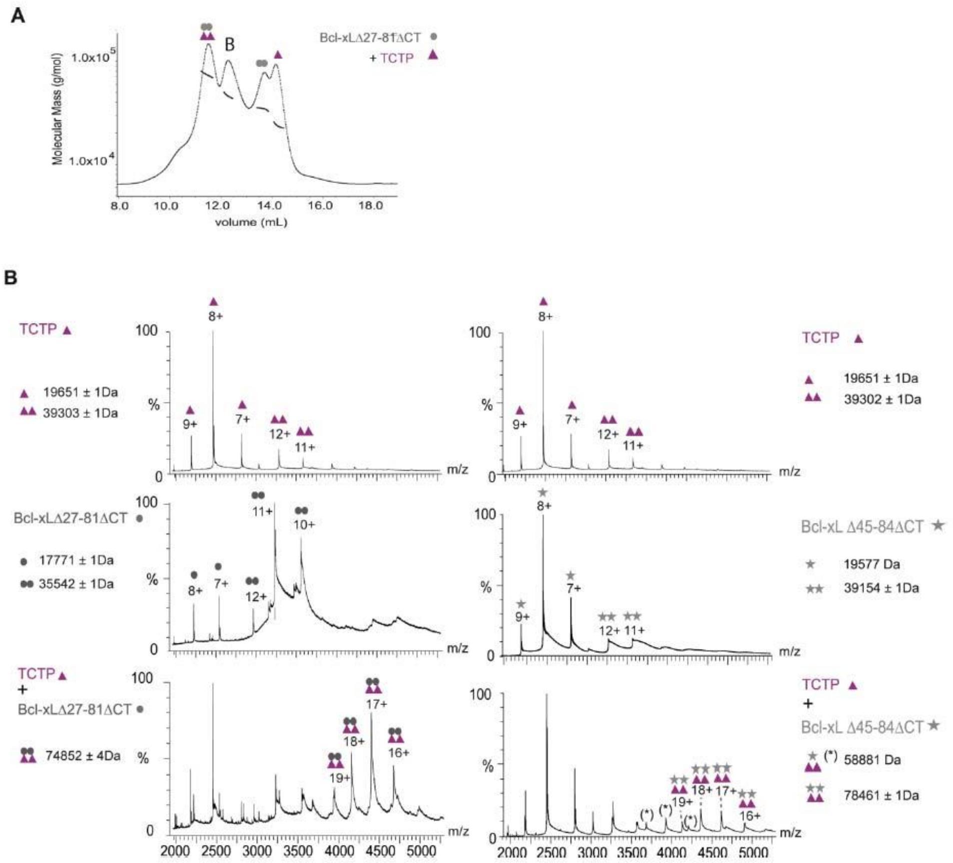

Supplementary Figure 4. Determination of TCTP:Bcl-xL (Δ27-81 ΔCT) complex stoichiometry. A) SEC-MALS analysis of TCTP+Bcl-xL(Δ27-81 ΔCT) (100μM),showing the elution profile with the direct molecular mass measurement of each elution peak. 3 populations are identified: a heterotetramer 2*TCTP:2*Bcl-xL(Δ27-81ΔCT) ( )eluting at 11.5ml with a measured molecular mass of 73.5 kDa ± 0.1%, a dimer of Bcl-xL(Δ 27-81 Δ CT) ( ) eluting at 13.8mL with a measured molecular mass of 36.1kDa ± 0.4%, and monomers of TCTP (▲) eluting at 14.1mL with a measured molecular mass of 20.9kDa ± 2.9%. **B**) Native mass spectra**, left panel** : TCTP (top, diluted at 5μM), Bcl-xL(Δ27-81-ΔCT) (middle, diluted at 5 μM) and TCTP+Bcl-xL(Δ27-81ΔCT) (down, diluted at 5 μM); Native mass spectra, right panel : Bcl-xL(Δ45-84 ΔCT)(middle, diluted at 5μM) and TCTP+Bcl-xL(Δ45-84ΔCT) (down, diluted at 5 μM). Populations and measured molecular masses are specified on the figure.

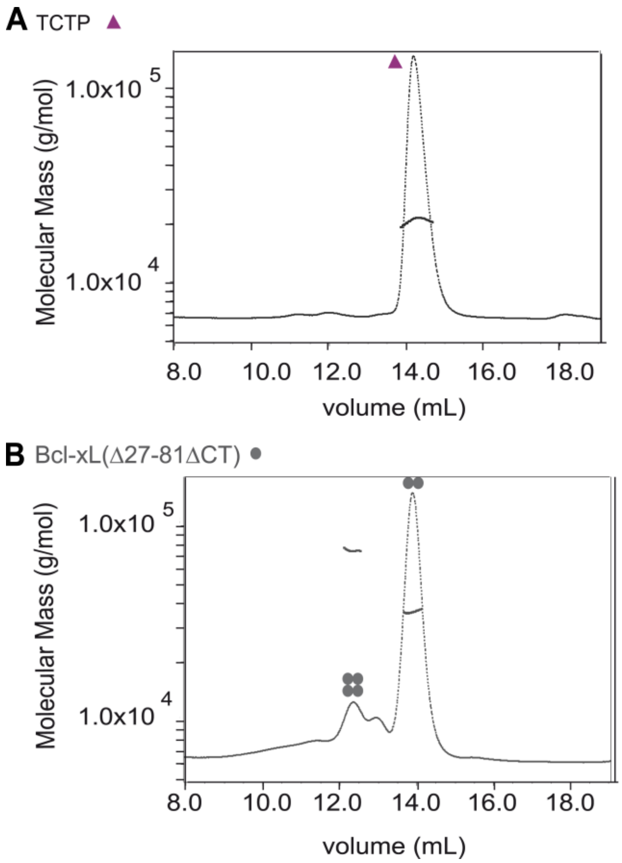

Supplementary Figure 5. SEC-MALS analysis of isolated proteins. A) SEC-MALS analysis of TCTP(100µM) showing the elution pro file with the direct molecular mass measurement of each elution peak. TCTP elutes as a monomer (▲) at 14.5mL with a measured molecular mass of 20.9kDa ± 2.9%; **B)** SEC-MALS analysis of Bcl-xL(Δ27-81ΔCT) (100µM). Bcl-xL(Δ27-81ΔCT) elutes mainly as a dimer (●●) at 14mL with a measured molecular mass of 36.1kDa ± 0.4% and as a small fraction of tetramers (⁘) at 12.4mL with a measured molecular mass of 74 kDa ± 0.6%.

Supplementary Table 1. Measured molecular weights in denaturing conditions

Sample	Theoretical molecular mass (Da)	Measured molecular mass (Da)	Associated species
TCTP	19651.4	19652.1 ± 0.3	monomer
	39302.8	39304.2 ± 0.4	dimer
TCTP-R21A	19790.5	19791.4 ± 0.1	monomer
	39581.0	39582.5 ± 0.3	dimer
Bcl-xL(Δ27-81ΔCT)	17770.8	17770.8 ± 0.4	monomer
Bcl-xL(Δ27-81ΔCT)-Y101K	17735.8	17735.6 ± 0.3	monomer
Bcl-xL(ΔCT)	23648.0	23648.9 ± 0.1	monomer
Bcl-xL(Δ45-84ΔCT)	19576.5	19577.3 ± 0.4	monomer

TCTP interacts with the BH3-groove of Bcl-xL

We then focused on the binding interface between TCTP and Bcl-xL. Since TCTP preferentially interacts with dimers of Bcl-xL we used the construct Bcl-xL(Δ27-81ΔCT) to analyze the behavior of two mutants, TCTP-R21A and Bcl-xL(Δ27-81ΔCT)-Y101K, described in literature for their ability to abolish interactions between TCTP and Bcl-2 family members[6,7].

The TCTP-R21A mutation was shown to specifically abrogate TCTP:Mcl-1 interactions[8]. To determine if the R21A mutation could also affect the binding between TCTP and Bcl-xL, we incubated TCTP-R21A with Bcl-xL(Δ27-81ΔCT) in an equimolar ratio (100µM) at 30°C, and checked the hetero-complexes formation by native MS (at 5µM). As shown in **Supplementary Figure 6B**, no peak corresponding to TCTP-R21A:Bcl-xL(Δ27-81ΔCT)hetero-complexes could be assigned. Therefore, residue R21 of TCTP is essential for its interaction with Bcl-xL, confirming that the N-terminal BH3-like domain of TCTP is involved in the TCTP:Bcl-xL interaction[9].

Except for p53, Bcl-xL interacts with its partners through its BH3 groove. To test whether this domain is involved in the interaction between TCTP and Bcl-xL, we used the Bcl-xL(Δ27-81ΔCT) -Y101K mutant. The Y101 residue, located at the C-terminus of helix $\alpha 2$, is part of the BH3-groove of Bcl-xL. Its mutation into lysine does not alter the folding of Bcl-xL but specifically abrogates its interaction with BH3 domains[6]**.** We thus incubated Bcl-xL(Δ27-81ΔCT)-Y101K and TCTP in an equimolar ratio (each protein at 100μM) at 30°C and analyzed the sample by native ESI-MS after a 5μM dilution (**Supplementary Figure 6C**). Here again, no hetero-complex was detected, strongly supporting that the Bcl-xL BH3-groove is implicated in the binding interface with TCTP.

To further corroborate the above results, we used the BH3-mimetic ABT-737, a novel anticancer molecule that specifically targets the BH3-groove of Bcl-2, Bcl-xL and Bcl-W proteins with high affinity but is unable to bind to Mcl-1[10,11]. Native ESI-MS experiments performed in the presence of TCTP, Bcl-xL(Δ27-81ΔCT) and a 10-fold molar excess of ABT-737 (**Supplementary Figure 6D**) revealed that the interaction between TCTP and Bcl-xL(Δ27-81ΔCT) is almost completely abrogated. As expected, ABT-737 was able to entirely displace TCTP from Bcl-xL (Δ27-81ΔCT), binding both monomers and dimers through a stoichiometry of 1 molecule per Bcl-xL (Δ27-81ΔCT) monomer. This experiment is fully consistent with the result of Bcl-xL(Δ27-81ΔCT)-Y101K and strongly suggests that TCTP interacts with the Bcl-xL BH3-groove.

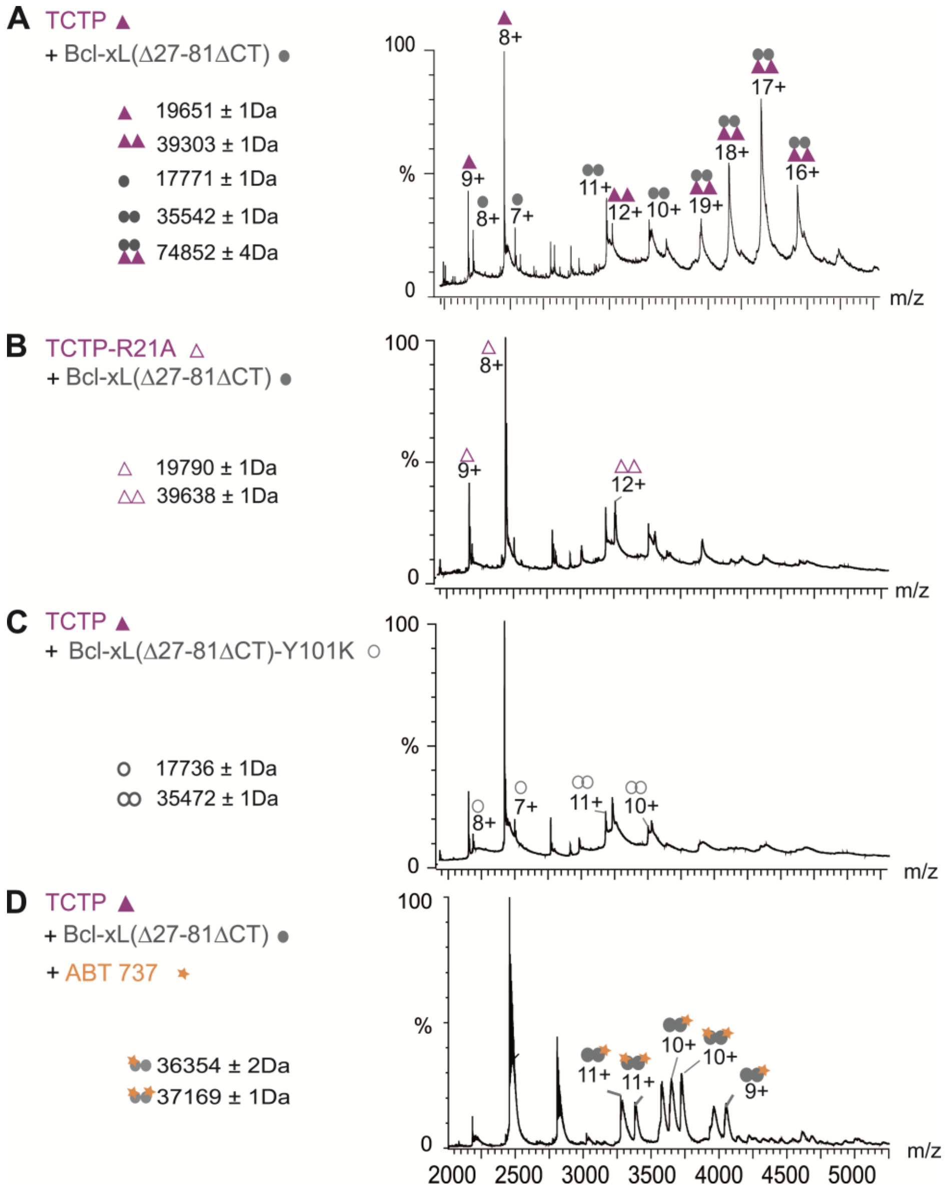

Supplementary Figure 6. Determination of the binding interface between TCTP and Bcl-xL(Δ27-81ΔCT) by native mass spectrometry. Native mass spectra of **A**) TCTP+Bcl-xL(Δ27-81ΔCT) (diluted at 5μM). **B**) TCTP-R21A+Bcl-xL(Δ27-81ΔCT) (diluted at 5μM), TCTP-R21A dimers exhibit an excess of 58Da compared to the expected mass due to the binding of a Cobalt atom (Co). **C**) TCTP+Bcl-xL(Δ27-81ΔCT)-Y101K (diluted at 5μM); **D**) TCTP+Bcl-xL(Δ27-81-ΔCT) (diluted at 5μM) + ABT737 (50μM). Populations and measured molecular masses are specified on the figure.

X-ray crystal structure of $TCTP_{11-31}$ in complex with Bcl-xL(Δ27-81ΔCT)

The full-length TCTP protein displays a unique fold containing three evolutionary conserved regions[1]: a central 9 stranded β-sheet including a short helix (α1), a helical hairpin formed by two helices (α2 and α3) and a long intrinsically unstructured loop connecting strands β5 and β6 (**Supplementary Figure 1A**). The TCTP BH3-domain encompasses the region between the strands β2 and β4. Considering that all known BH3 domains fold into an α-helix, it was of interest to determine whether TCTP-BH3 domain folds into an α-helix or a β-sheet while interacting with Bcl-xL. However, some BH3 domains may have been misidentified in a number of proteins, presumably because of the low complexity of the consensus sequence[12]. This prompted us to examine the interaction between TCTP-BH3 domain and Bcl-xL in more detail. Accordingly, we determined the structure of a deletion variant of Bcl-xL(Bcl-xLΔ27-81ΔCT) in complex with TCTP BH3 domain ($TCTP_{11-31}$) using X-Ray crystallography (see main text and **Table 1**).

Overall structure of Bcl-xL: TCTP-BH3 domain complex

As observed in other complexes using the deleted form of Bcl-xL, the asymmetric unit contains a dimeric form of Bcl-xL that exhibits a swapping domain, exchanging their α1-helix (**Supplementary Figure 7**). This kind of swapping domain is considered to be a crystallization artifact due to the Δ27-81 deletion, but it doesn't affect the BH3-domain-binding affinity or anti-apoptotic properties of the protein. This swapping domain was also observed in the WT vaccinia virus F1L, a Bcl-2-like protein lacking the loop between α1 and α2[4]. Unexpectedly, the truncated form of Bcl-xL in complex with TCTP-BH3 peptide exhibits two binding sites. The first binding interface exhibits the same interactions observed in the asymmetric unit of Bcl-xL in complex

with the binding BH3 domain of SOUL[13], Beclin-1[14] and BimSAHB[15]. The second binding site located at the BclxL dimer interface, containing two TCTP-BH3 peptides linked by an internal disulfide bridge involving the two C28 residues, is most probably specific for the crystallization process and not physiologic. Indeed, this interface does not involve the Bcl-xL-Y101 residue which specifically abrogates Bcl-xL/TCTP-BH3 interactions and does not reflect the 1:1 ratio observed during ITC experiments, SEC-MALS and native MS experiments.

The two monomers present in the asymmetric unit of the crystal of the TCTP-BH3 complex are very similar with a rmsd (root mean square deviation) of 0.185 Å over 433 backbone atoms of Bcl-xL and the TCTP-BH3 domain. Irrespective of the swapping domain, each monomeric unit aligns extremely well with Bcl-xL (rmsd=0.4 Å over 407 backbone atoms), Bcl-xL-BeclinBH3 (rmsd=0.35 Å over 418 backbone atoms) and Bcl-xL-BimSAHB (rmsd=0.42 Å over 342 backbone atoms). This suggests that the overall fold and more importantly the BH3 binding groove remains intact during crystallization. Therefore, the interaction between the TCTP BH3 fragment and Bcl-xL likely reflects their normal mode of interaction, unaffected by the swapping domain occurring on the opposite side of molecule.

Overall, the (Bcl-xL(Δ27-81ΔCT)/TCTP-BH3) structure is similar to ones previously determined and involving Bcl-xL in complex with Bak, Bad, Bim, PUMA, Beclin and SOUL peptides (**Supplementary Figure 7**)[13-19]. Each heterodimeric unit, composed of the first helix of BclxL chain B (residues 1-26), Bcl-xL chain A (residues 83-206) and one TCTP BH3 peptide, forms a compact globular form. As seen in all other Bcl-xL/BH3 domain complexes, Bcl-xL contains eight α -helices (α1- α8) among which the completely hydrophobic α5, surrounded by seven shell helices (α1-α4 and α6-α8). A hydrophobic groove created by α2-α5 provides the docking surface

for the TCTP-BH3 peptide. In this binding site, on the 20 amino-acids of TCTP-BH3 peptide co-crystallized with Bcl-xL, only 11 (amino-acids 15 to 26) show clear electron densities and adopt a four turn α-helix in the complex, confirming our prediction that TCTP is a BH3 domain-only Bcl-xL-binding protein.

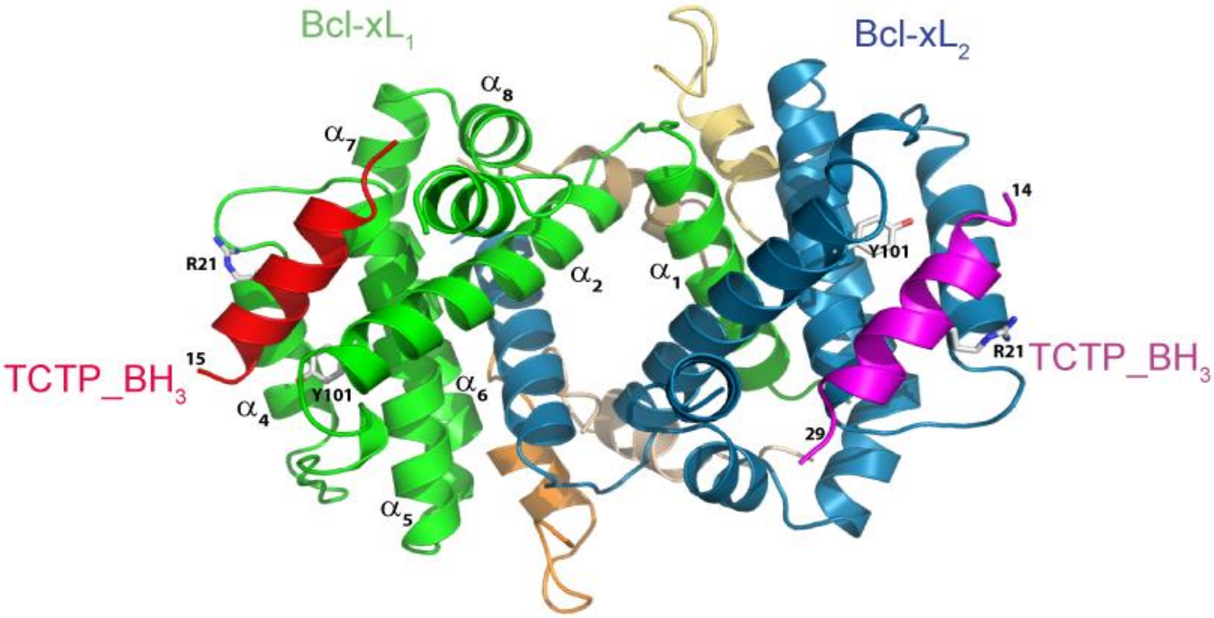

Supplementary Figure 7. Crystal structure of Bcl-xL:TCTP-BH3 complex. Cartoon representation of truncated Bcl-xL bound to TCTP-BH3 domain. Truncated Bcl-xL crystallises in a dimeric form (green and blue) with a helix α1 swapping. For each monomer of Bcl-xL one TCTP-BH3 peptide binds its BH3 groove (red and magenta) and two dimers of TCTP-BH3 peptide bind at the interface of Bcl-xL dimer.

Interface of Bcl-xL:TCTP-BH3 domain complex

The interaction between Bcl-xL and the BH3 domain of TCTP is mediated by both hydrophobic and electrostatic interactions (see **Fig. 2 A,B and Supplementary Figure 8A**). As in other Bcl-xL/BH3 domain complexes, side chains of the hydrophobic residues at the P2(I20), P3(I23) and P4(L27) positions form a network of van der Waals interactions with the Bcl-xL groove. Three additional residues, I17, L29 and the side chain of K19, participate in this hydrophobic packing. I17, positioned in P1+1, might counterbalance the lack of the usual P1 position. They pack with the hydrophobic residues F97, Y101, F105, L108, V126, V141, F146 and Y195 of Bcl-xL in the target-binding pocket formed by α2-α5 helices. Several flanking hydrogen bonds stabilize the interaction; they involve the R21 residue of the TCTP BH3 domain in interaction with E129 and D133 in Bcl-xL, and the D25 residue of the BH3 domain signature, in interaction with R139 in Bcl-xL.

The amino acid contribution to the free energy of BH3 peptides: Bcl-xL binding has been calculated using molecular dynamics simulations coupled to molecular mechanics/Poisson-Boltzmann surface area methods[20]. The Bcl-xL residues that give important contributions are: F97, Y101, L112, V126, L130, R139, F146 and Y195. With the exception of L112 and L130, all these residues participate in the interaction with TCTP-BH3 peptide. It is likely that the lack of these 2 important contributors affects the interaction and contributes to the lower binding affinity observed for the TCTP-BH3 peptide in comparison with other BH3-only peptides.

Proteins rearrangement upon binding

A comparison between the crystal structures of unbound Bcl-xL and Bcl-xL/TCTP-BH3 domain complex reveals similar structural rearrangements as seen in other Bc-xL/BH3 domain complexes (**Supplementary Figure 8B**). Upon binding to the TCTP BH3 domain, the N-terminal part of Bcl-xL α4 helix moves toward the peptide by ~5-6Å, whereas α3 partially melts and opens up to 5Å. Another strong conformational change upon binding occurs in helix α3 and the loop connecting helices α2–α3. In the apo-form, Bcl-xL residues F105 in helix α3 and Y101 in the α2-α3 loop, are packed with the rest of the hydrophobic residues in the BH3-groove pocket. Upon binding, helix α2 elongates, accompanied by the flipping of F105 outwards to form a hydrophobic groove that can accommodate the binding of the TCTP BH3 peptide, while Y101 is completely buried into the core hydrophobic interface with the TCTP BH3 peptide. Interestingly, among all other Bcl-xL/BH3 domain structures, only Beclin 1 and Bak BH3 peptides show such reorganizations[14,16,19,21]. The most striking rearrangement observed concerns the β-sheet to α-helix switch of TCTP (amino acids 15-29). In the free full length TCTP protein, residues 15 to 29 include the C terminus of the strand β2, strand β3, the turn β3- β4 and the N-terminus of strand β4, which are packed with other β strands and the two α-helices to maintain the hydrophobic core of TCTP. Upon binding to Bcl-xL, the TCTP BH3 peptide folds as a 3 turns α-helix (**Supplementary Figure 7, Supplementary Figure 8A, B**). Interestingly, the hydrophobic TCTP BH3 domain residues involved in the interface with Bcl-xL are not exclusively present in the hydrophobic core of the β barrel of isolated TCTP protein: I17, I20 and I23 are solvent exposed, whereas L27and L29 are completely buried within the hydrophobic core of TCTP (**Supplementary Figure 9)**. Charged residues R21 and D25, which are involved in a salt-bridge network with Bcl-xL BH3 groove, are fully solvent exposed.

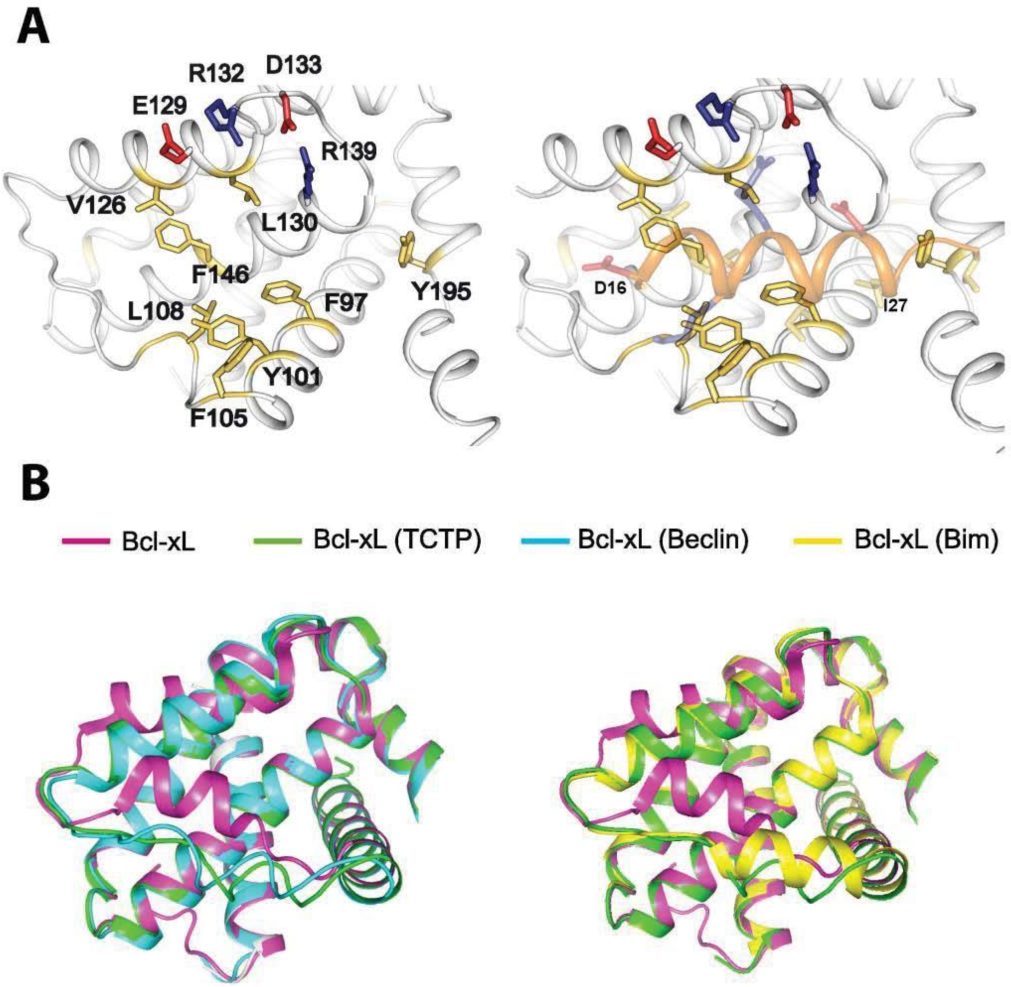

Supplementary Figure 8. TCTP-BH3 peptide interacts with the Bcl-xL groove and induces structural rearrangements. A) Cartoon representation of Bcl-xL:TCTP-BH3 complex including (right panel) or not TCTP (left panel). Bcl-xL is colored in grey, TCTP-BH3 is colored in orange. **B)** Cartoon representation of Bcl-xL in complex or not with different BH3 domains: Left panel, superimposition of Bcl-xL alone (pink), in complex with Beclin-BH3 peptide (cyan) or TCTP-BH3 peptide (green); Right panel superimposition of Bcl-xL alone (pink), in complex with Bim-BH3 peptide (yellow) or TCTP-BH3 peptide (green).

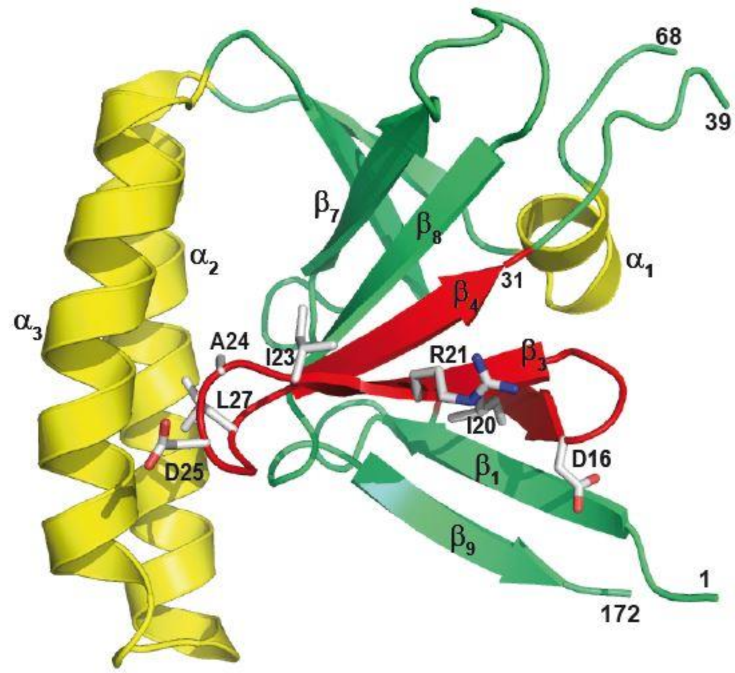

Supplementary Figure 9. Cartoon representation of TCTP structure (PDB code: 1YZ1). The putative BH3 domain is coloured in red. Conserved residues in BH3 domains are represented in sticks. The residues I20, I23 and L27 correlate with the position of conserved hydrophobic BH3 domains residues P2, P3 and P4. R21 correlates with one conserved charged residues in BH3 domains. A24 and D25 count for the GD signature in BH3 domains.

Inhibition of membrane permeablization

The TCTP peptides potentiate Bcl-xL for inhibition of membrane permeablization in an *in vitro* liposome system. **Supplementary Figure 10** shows the results of the same experiment as described in **Fig. 3** of the main text but using the TCTP peptide instead of the full-length protein. Bcl-xL was co-incubated with different molecular ratios [XL:TCTP] of TCTP (1-31) WT peptides (**Fig. 10 A,C**) or fixed amounts of TCTP (1-31) R21A peptides (**Supplementary Figure**

10 B,D) at pH 9 for 45 min at 37 degrees. The treated Bcl-xL was then titrated into reactions containing ANTS-DPX liposomes, 100nM Bax and 20nM of either cBid (**Supplementary Figure 10 A,B**) or cBid mt1 (**Supplementary Figure 10 C,D**) at pH 7 to quantify the activity of Bcl-xL inhibition of liposome permeablization. As a control, 3.2μM pH9 treated $TCTP_{1\text{-}31}$ (equal to the maximum amount added to the liposome system in **Supplementary Figure 10 A-D** was added to the system containing ANTS-DPX liposomes, 100nM Bax and 20nM of cBid or cBidmt1 (**Supplementary Figure 10 E**). The permeablization of liposomes was indicated by the relative release percentage (%) of entrapped fluorescent dye ANTS and collisional quencher DPX in end-point assays after 5h incubation at 37 degree compared to full release by adding detergent (1% Triton X-100). Data represent average values from 3 independent repeats and error bars indicate standard deviation. For statistical analysis see **Method summary and statistical analysis**.

Supplementary Figure 10

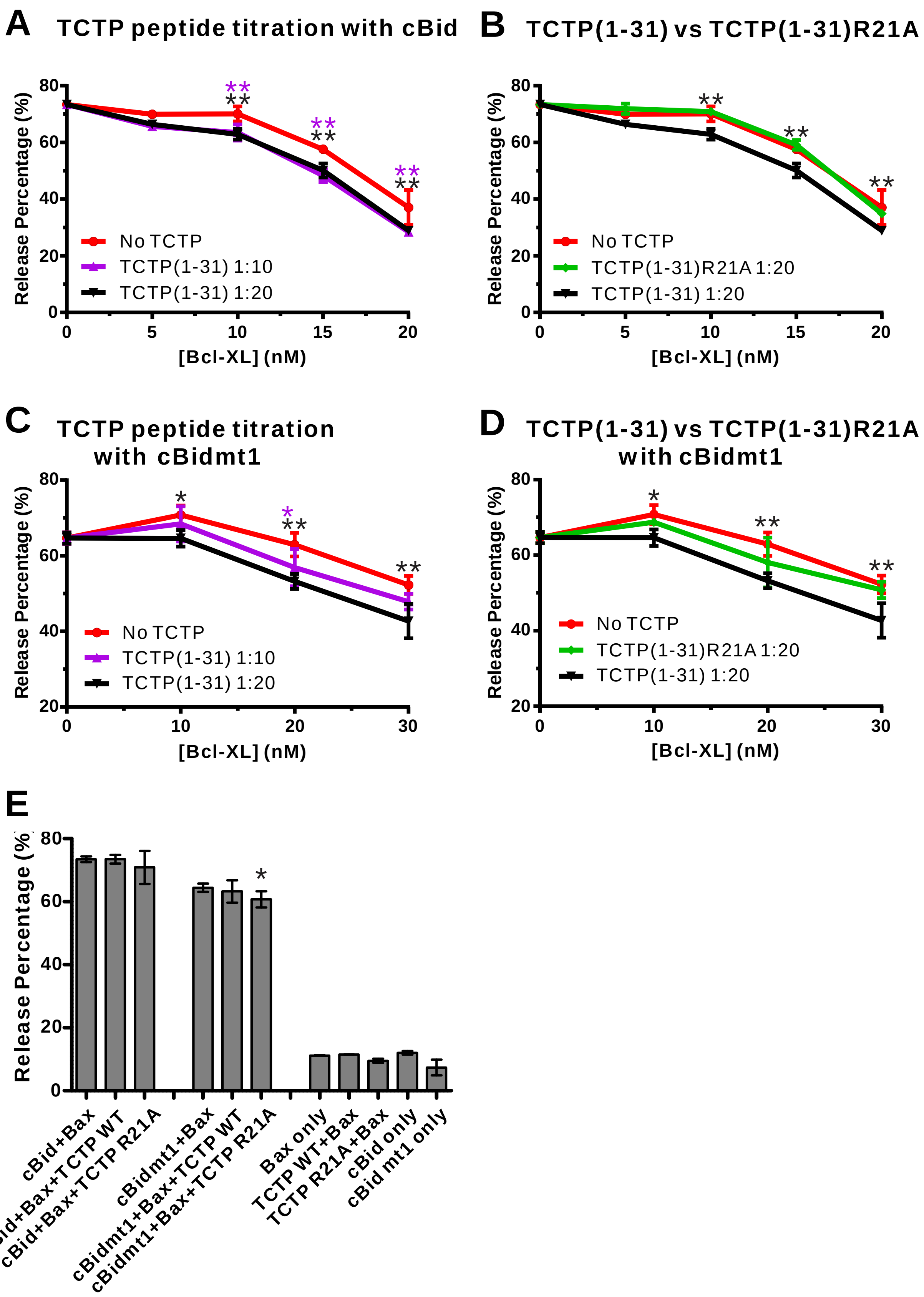

Supplementary Figure 10.

TCTP11-31 potentiates Bcl-xl inhibition of Bid/Bax mediated liposome permeabilization.

The TCTP peptides potentiate Bcl-xL for inhibition of membrane permeabilization in an *in vitro* liposome system. **Supplementary Figure 10** shows the results of the same experiment as described in **Fig. 3** of the main text but using the TCTP peptide instead of the full-length protein. Bcl-xL was co-incubated with different molecular ratios [XL:TCTP] of TCTP (1-31) WT peptides (**Fig. 10 A,C**) or fixed amounts of TCTP (1-31) R21A peptides (**Supplementary Figure 10 B,D**) at pH 9 for 45 min at 37 degrees. The treated Bcl-xL was then titrated into reactions containing ANTS-DPX liposomes, 100nM Bax and 20nM of either cBid (**Supplementary Figure 10 A,B**) or cBid mt1 (**Supplementary Figure 10 C,D**) at pH 7 to quantify the activity of Bcl-xL inhibition of liposome permeabilization. As a control, 3.2μM pH9 treated $TCTP_{1-31}$ (equal to the maximum amount added to the liposome system in **Supplementary Figure 10 A-D** was added to the system containing ANTS-DPX liposomes, 100nM Bax and 20nM of cBid or cBidmt1 (**Supplementary Figure 10 E**). The permeabilization of liposomes was indicated by the relative release percentage (%) of entrapped fluorescent dye ANTS and collisional quencher DPX in end-point assays after 5h incubation at 37 degree compared to full release by adding detergent (1% Triton X-100). Error bars, std. dev. n=3. To compare between the TCTP peptide treated groups and the Control group statistically, two-way ANOVA test was performed for **A-D** and one-way ANOVA test was performed for **E**. Dunnett's multiple comparisons test was used to calculate the significance of difference between the TCTP peptide treated groups and the corresponding control group (No TCTP for **A-D**, cBid+Bax or cBidmt1+Bax for **E**). Colored asterisks above each data point indicate the statistical significance (*:$p<0.05$; **:$p<0.01$,no asterisks if $p\geq0.05$). A complete statistical analysis is provided in **Supplementary Table 2-4**.

Supplementary Figure 11

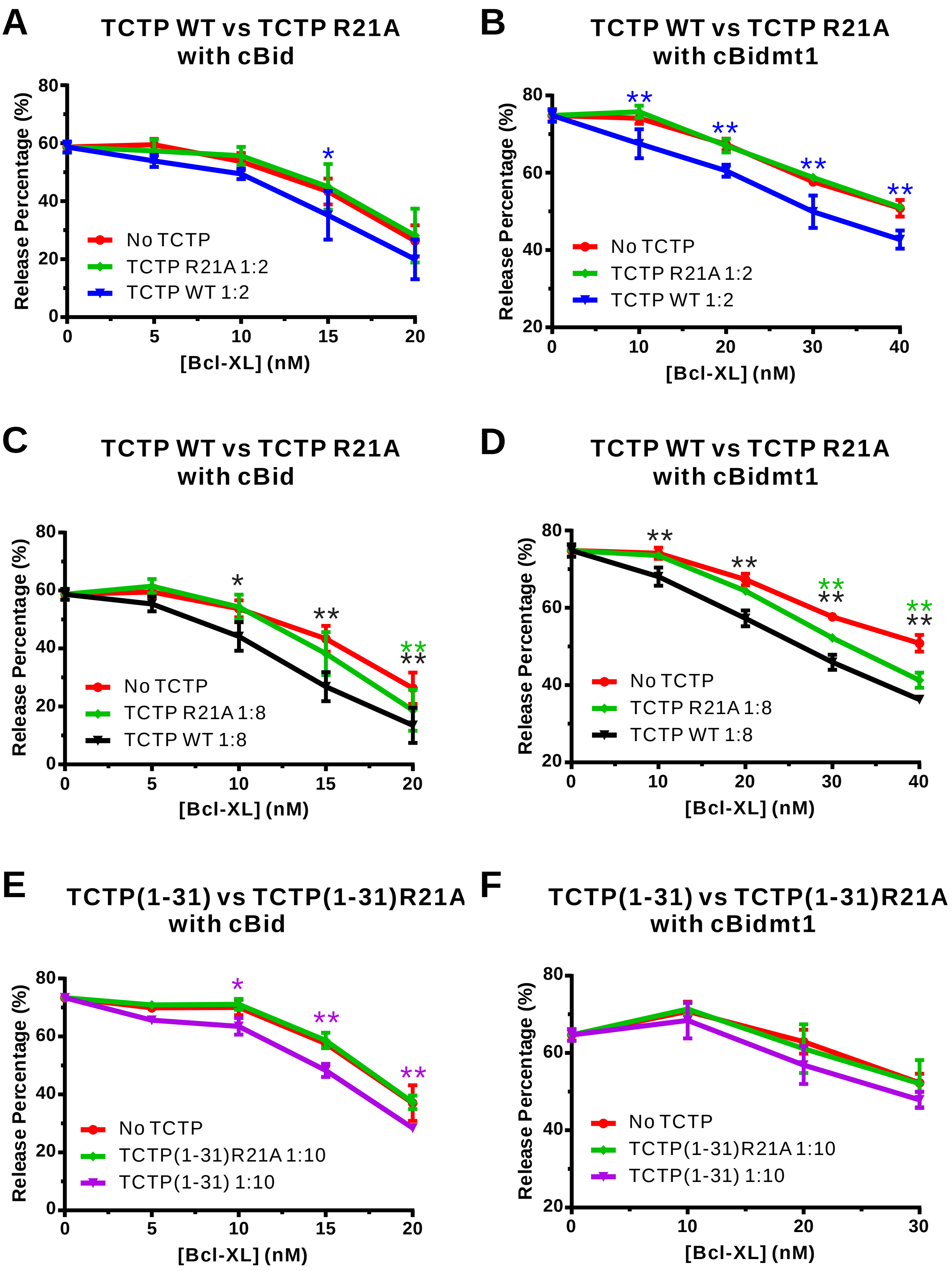

Supplementary Figure 11:

A-D. *in vitro* reconstitution assay on liposome permeabilization. Increasing concentrations of Bcl-xL were incubated in with a fixed 1:2(**A, B**) or 1:8 (**C, D**) molecular ratios [Bcl-xL:TCTP] with TCTP WT or TCTP R21A at pH 9 for 45 min at 37°C, as specified. The treated Bcl-xL was then added to reactions containing ANTS-DPX liposomes, 100nM Bax and 20nM of either cBid (**A, C**) or cBid mt1(**B, D**) at pH 7 and liposome permeabilization was quantified by fluorescence after 5h incubation at 37°C where 100% release was defined as the fluorescence change due to lipid solubilization with 1% Triton X-100.

E-F. Bcl-xL was co-incubated with 1:10 molecular ratio [XL:TCTP] of TCTP (1-31) WT peptides or TCTP (1-31) R21A peptides at pH 9 for 45 min at 37 degrees. The treated Bcl-xL was then titrated into reactions containing ANTS-DPX liposomes, 100nM Bax and 20nM of either cBid (**E**) or cBid mt1 (**F**) at pH 7 to quantify the activity of Bcl-xL inhibition of liposome permeabilization as described in **A-D**.

Error bars, std. dev. n=3. Two-way ANOVA test was performed to compare different groups and further comparison between the TCTP protein or TCTP peptides treated groups and the Control group (No TCTP) were analyzed through Dunnett's multiple comparisons test at each sample condition (Bcl-XL concentration) for statistical significance. Colored asterisks above each data point indicate the statistical significance (*:$p<0.05$; **:$p<0.01$, no asterisks if $p\geq0.05$). A complete statistical analysis is provided in **Supplementary Table2-4**.

Method summary and statistical analysis: For Figure 3A-D and supplementary Figure 10 A-D, S14, two-way ANOVA test was performed to compare different groups (e.g. No TCTP, TCTP 1:4 etc.) and further comparison between the TCTP protein or TCTP peptides treated groups and the Control group (No TCTP) were analyzed through Dunnett's multiple comparisons test at each sample condition (Bcl-XL concentration) for statistical significance. NOTE: the first point ([Bcl-XL]=0nM) are values from same wells, so P value is always equal to 1

For Figure 3E and supplementary Figure 10E, one-way ANOVA test was performed to compare the TCTP the TCTP protein or TCTP peptides treated groups and the Control group (cBid+Bax or cBidmt1+Bax) to test if TCTP inhibit Bax activation directly. Statistical significance was analyzed through Dunnett's multiple comparisons test.

Yellow highlighted ones are significant value ($p<0.05$ aka 95% confidence)

p value test tables:

Supplementary Table 2:

	[Bcl-XL] (nM)	0	5	10	15	20
Figure 3A	No TCTP vs. TCTP WT 1:2	1.00	0.08	0.24	0.00	0.04
	No TCTP vs. TCTP WT 1:4	1.00	0.14	0.06	< 0.01	< 0.01
	No TCTP vs. TCTP WT 1:8	1.00	0.25	< 0.01	< 0.01	< 0.01
Figure 3B	No TCTP vs. TCTP R21A 1:4	1.00	0.72	0.67	0.68	0.52
	No TCTP vs. TCTP WT 1:4	1.00	0.16	0.08	< 0.01	< 0.01
Sup Figure 10A	No TCTP vs. TCTP(1-31) 1:10	1.00	0.05	0.01	< 0.01	< 0.01
	No TCTP vs. TCTP(1-31) 1:20	1.00	0.13	< 0.01	< 0.01	< 0.01
Sup Figure 10B	No TCTP vs. TCTP(1-31)R21A 1:20	1.00	0.45	0.87	0.62	0.40
	No TCTP vs. TCTP(1-31) 1:20	1.00	0.11	< 0.01	< 0.01	< 0.01
Sup Figure 14A	No TCTP vs. TCTP R21A 1:2	1.00	0.68	0.71	0.81	0.74
	No TCTP vs. TCTP WT 1:2	1.00	0.10	0.25	0.01	0.06
Sup Figure 14C	No TCTP vs. TCTP R21A 1:8	1.00	0.65	0.96	0.08	0.01
	No TCTP vs. TCTP WT 1:8	1.00	0.19	< 0.01	< 0.01	< 0.01
Sup Figure 14E	No TCTP vs. TCTP(1-31)R21A 1:10	1.00	0.83	0.80	0.81	0.99
	No TCTP vs. TCTP(1-31) 1:10	1.00	0.06	0.01	< 0.01	< 0.01

Supplementary Table 3:

	[Bcl-XL] (nM)	0	10	20	30	40
Figure 3C	No TCTP vs. TCTP WT 1:2	1.00	< 0.01	< 0.01	< 0.01	< 0.01
	No TCTP vs. TCTP WT 1:4	1.00	0.08	< 0.01	< 0.01	< 0.01
	No TCTP vs. TCTP WT 1:8	1.00	< 0.01	< 0.01	< 0.01	< 0.01
Figure 3D	No TCTP vs. TCTP R21A 1:4	1.00	0.93	0.52	0.10	0.07
	No TCTP vs. TCTP WT 1:4	1.00	0.01	< 0.01	< 0.01	< 0.01
Sup Figure 10C	No TCTP vs. TCTP(1-31) 1:10	1.00	0.56	0.05	0.17	
	No TCTP vs. TCTP(1-31) 1:20	1.00	0.04	< 0.01	< 0.01	
Sup Figure 10D	No TCTP vs. TCTP(1-31)R21A 1:20	1.00	0.63	0.11	0.79	
	No TCTP vs. TCTP(1-31) 1:20	1.00	0.04	< 0.01	< 0.01	
Sup Figure 14B	No TCTP vs. TCTP R21A 1:2	1.00	0.53	0.97	0.75	0.99
	No TCTP vs. TCTP WT 1:2	1.00	< 0.01	< 0.01	< 0.01	< 0.01
Sup Figure 14D	No TCTP vs. TCTP R21A 1:8	1.00	0.86	0.06	< 0.01	< 0.01
	No TCTP vs. TCTP WT 1:8	1.00	0.00	< 0.01	< 0.01	< 0.01
Sup Figure 14F	No TCTP vs. TCTP(1-31)R21A 1:10	1.00	0.97	0.78	1.00	
	No TCTP vs. TCTP(1-31) 1:10	1.00	0.65	0.10	0.26	

Supplementary Table 4:

Figure 3E	cBid+Bax vs. cBid+Bax+TCTP WT	0.57
	cBid+Bax vs. cBid+Bax+TCTP R21A	0.11
	cBidmt1+Bax vs. cBidmt1+Bax+TCTP WT	0.85
	cBidmt1+Bax vs. cBidmt1+Bax+TCTP R21A	0.95
Sup Figure 10E	cBid+Bax vs. cBid+Bax+TCTP(1-31) WT peptide	1.00
	cBid+Bax vs. cBid+Bax+TCTP(1-31) R21A peptide	0.60
	cBidmt1+Bax vs. cBidmt1+Bax+TCTP(1-31) WT peptide	0.76
	cBidmt1+Bax vs. cBidmt1+Bax+TCTP(1-31) R21A peptide	0.05

Supplementary Figure 12.

Anti-apoptotic effect of Bcl-xL against Bax at the mitochondrial level in a reconstituted system using identical conditions as in Fig. 3F but now instead of the proteins using peptides (in blue instead of the red used in the main text for the protein).

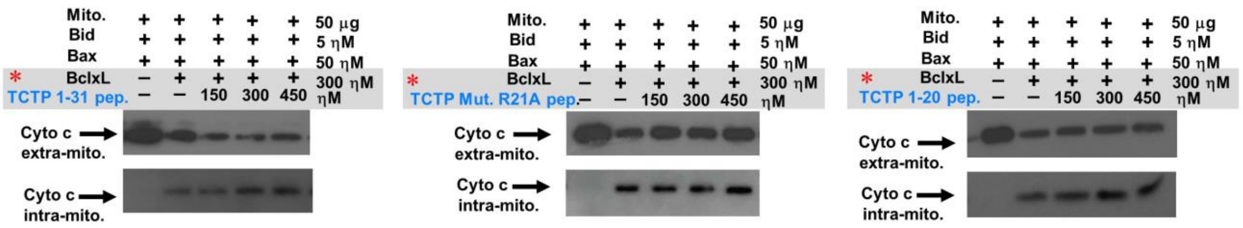

Supplementary Figure 12. Permeabilization assays of the outer mitochondrial membrane**.** See legend of Figure 3F**.**

Permeabilization assays[22] of the outer membrane of freshly isolated mouse liver mitochondria by tBid and Bax assessing cytochrome c (Cyto c) release by Western Blot analysis. In these experiments sub-optimal amounts of Bcl-xL were added as to only partially inhibit Bax mediated cytochrome *c* release. Bcl-xL and variants of TCTP peptide were all pre-incubated at µM concentrations (TCTP ranging from 1.5-6µM and Bcl-xL 3µM, framed in gray with red *asterisk to highlight the pre-incubation*), pH 9, 30 C° before being added to the mitochondria at their final concentrations. 50µg of mitochondrial proteins (Mito.) were then incubated at 30 C° for 15 min. in the presence of 5nM Bid, 50nM Bax, 300nM Bcl-xL (+) and TCTP1-31 peptide or mutant R21A of TCTP1-31 peptide or TCTP1-20 peptide at the indicated concentrations. Cytochrome c was detected in the mitochondrial fraction (Cyto c intra-mito) or in the supernatant (Cyto c extra-mito) by Western blot analysis.

Supplementary Figure 13. Rescue of TCTP $^{+/-}$ haploinsufficiency and sensitivity to γ-irradiation induced apoptosis by WT-TCTP protein and peptide but not by the mutant protein and peptides

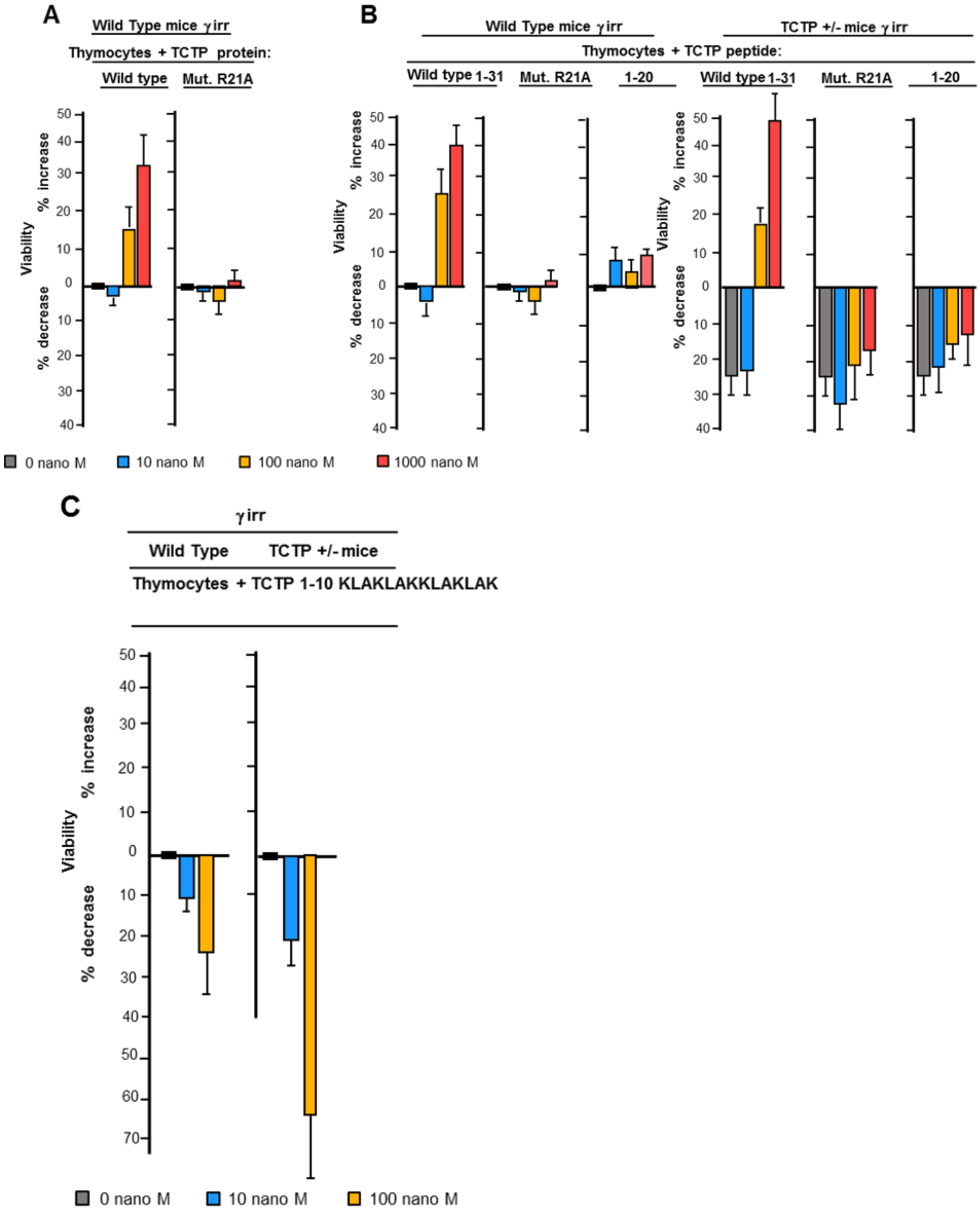

D

peptide TCTP-KLA nanoM	% Viability: γ-irradiated thymocytes: wild type	TCTP$^{+/-}$	Cell lines from Hyo Young Kim et al.(2011): A549	ACHN	AGS	HeLa	HepG2	SK-Hep1
10	90%	80%						
100	75%	35%	80%	90%	85%	100%	110%	100%
1000			85%	95%	85%	100%	110%	100%
10000			65%	50%	20%	60%	80%	80%

Supplementary Figure 13. A) Thymocytes from WT mice were γ-irradiated (γ-irr) as in described for **Fig. 3G** in the main text and cultured in the presence of the TCTP proteins. **B)** Thymocytes from WT and TCTP$^{+/-}$ mice were irradiated and cultured in the presence of the peptide instead of the protein (WT TCTP peptide 1-31, mutant TCTP peptide bearing the mutation R21A, or peptide 1-20 at the indicated concentrations). **C)** Positive control using a peptide with the 10 first amino acids of TCTP coupled to an apoptotic signaling sequence (KLA). (**D**) Comparison between thymocytes freshly derived from mice as presented in this study and cell lines in culture for their cell death after transduction with a TCTP peptide bound to KLA. Typically KLA is inducing apoptosis. Viability (%) of cells after treatment with a peptide TCTP-KLA in γ-irradiated thymocytes and cell lines described before[23].

Supplementary Figure 14.

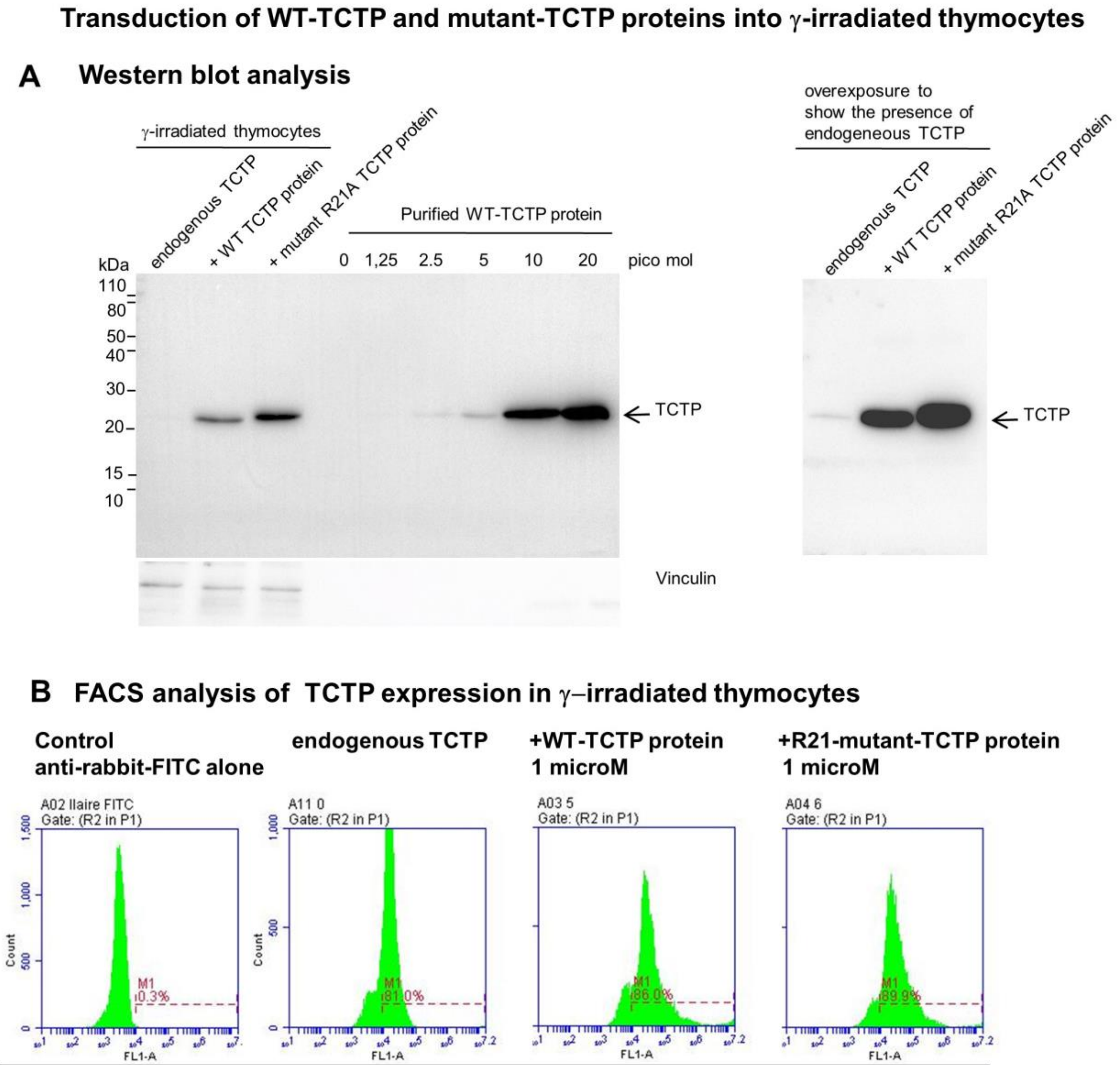

**% positive cells:**	**0.3%**	**81.0%**	**86.0%**	**89.9%**
**Mean intensity:**	**2 837**	**47.829**	**274.762**	**165.144**

C **FACS analysis of TCTP-FLAG peptides of γ–irradiated thymocytes using rabbit anti-FLAG and anti-rabbit-FITC**

No peptide	TCTP-WT-peptide 10 microM	TCTP-R21 mutant-peptide 10 microM	TCTP/Bax-hybrid-peptide 10 microM

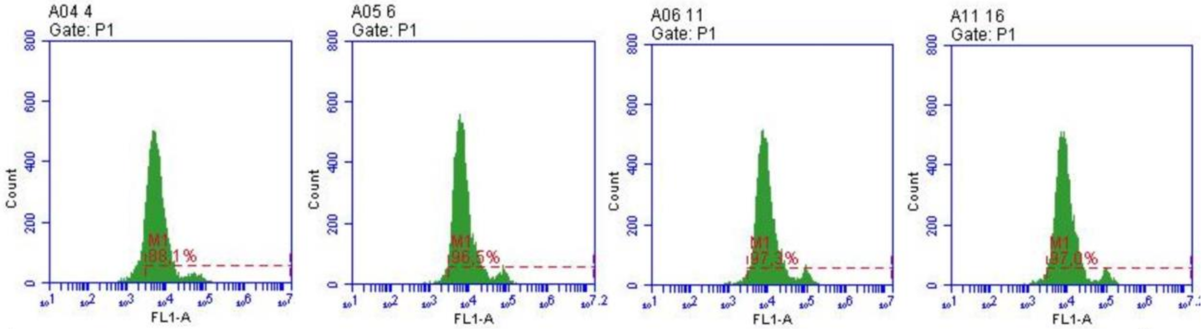

% positive cells:	88.1%	96.5%	97.3%	97.0%
Mean intensity:	9 096	12 033	16 046	16 327

Control anti-rabbit-FITC alone	TCTP-WT-peptide 50 microM	TCTP-R21 mutant-peptide 50 microM	TCTP/Bax-hybrid-peptide 50 microM

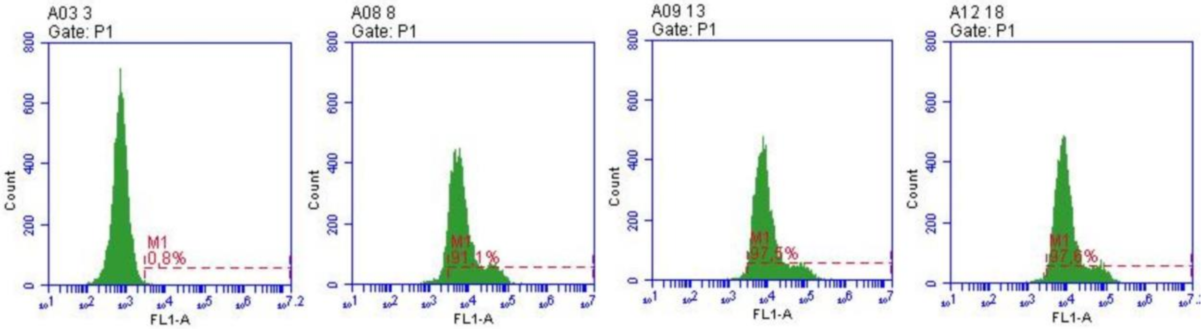

% positive cells:	0.8%	91.1%	97.5%	97.6%
Mean intensity:	824	14 425	19 033	18 355

TCTP-WT-peptide 100 microM	TCTP-R21 mutant-peptide 100 microM	TCTP/Bax-hybrid-peptide 100 microM

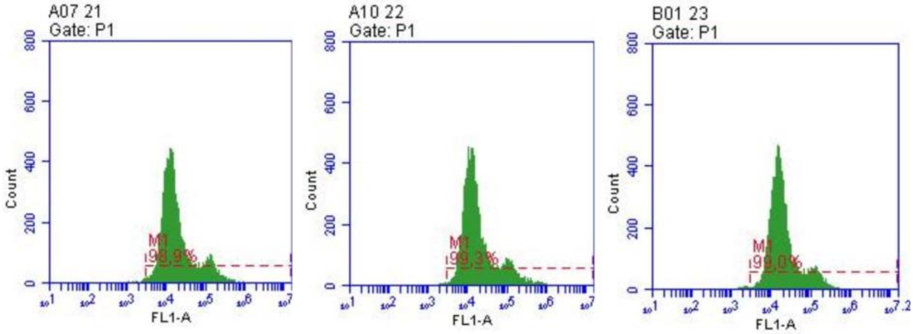

% positive cells	98.9%	99.3%	99.0%
Mean intensity:	45 080	46 196	38 103

D **FACS analysis of TCTP of γ– irradiated thymocytes using direct labeling with anti-FLAG-phyco-erythrin**

No peptide | **TCTP-WT-peptide 10 microM** | **TCTP-R21 mutant-peptide 10 microM** | **TCTP/Bax-hybrid-peptide 10 microM**

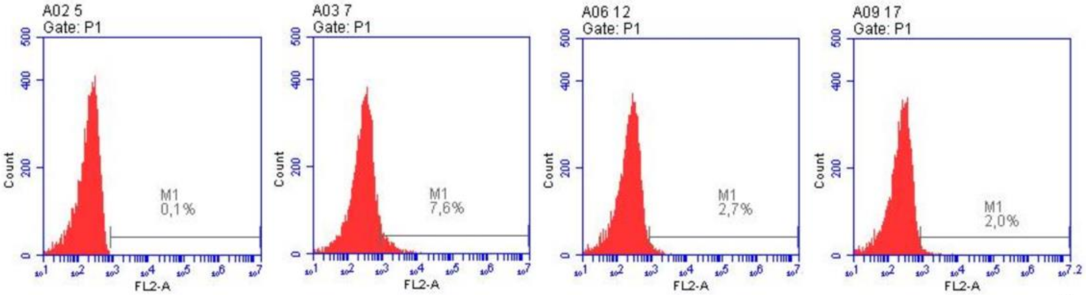

**% positive cells:**	**0.1%**	**7.6%**	**2,7%**	**2,0%**
**Mean intensity:**	**206**	**403**	**421**	**1 273**

TCTP-WT-peptide 50 microM | **TCTP-R21 mutant-peptide 50 microM** | **TCTP/Bax-hybrid-peptide 50 microM**

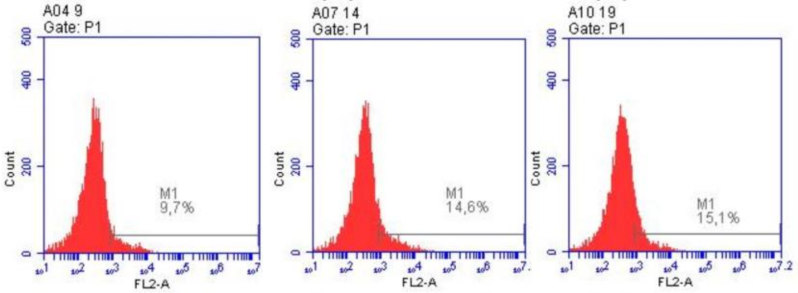

**% positive cells:**	**9.7%**	**14.6%**	**15.1%**
**Mean intensity:**	**544**	**775**	**671**

TCTP-WT-peptide 100 microM | **TCTP-R21 mutant-peptide 100 microM** | **TCTP/Bax-hybrid-peptide 100 microM**

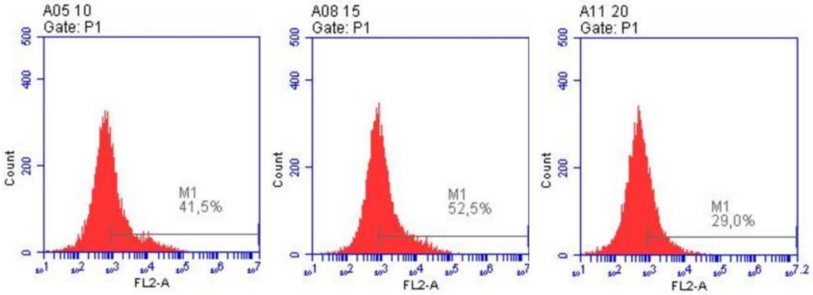

**% positive cells:**	**41.5%**	**52.5%**	**29.0%**
**Mean intensity:**	**2 743**	**3 034**	**1 101**

Supplementary Figure 14. Transduction of WT and mutant TCTP proteins and peptides into γ-irradiated thymocytes. A) Incorporation of exogenous recombinant TCTP full length protein into γ-irradiated thymocytes. Western blot analysis of γ-irradiated thymocytes incubated without adding any exogenous protein (first lane), with the full length recombinant WT TCTP protein (second lane) or with R21A-mutant (third lane) TCTP protein. Increasing amounts of full length WT TCTP protein (lanes 5-9). A longer exposure of the three first lanes is displayed in the right panel. Polyclonal rabbit anti-TCTP antibodies are used to detect TCTP. Vinculin is used as equal loading. **B)** FACS analysis for the same experiment. **C)** FACS analysis of the transduction of WT-TCTP-FLAG peptides, R21-mutant TCTP-FLAG peptides and TCTP/Bax-hybrid-FLAG peptides into γ-irradiated thymocytes. Concentrations 10 μM (upper panels), 50 μM (middle panels) and 100 μM (lower panels) and rabbit anti-FLAG and anti-rabbit-FITC were used. **D)** FACS analysis of the same experiment showed in D. The comments for these results are in the main text. The data presented in this Supplementary figure 14 show a representative experiment performed in triplicate were the standard deviation did not exceed 7%.

Supplementary Methods

Constructs expression and mutagenesis

For expression as a GST-fusion protein, TCTP was cloned into a pHGGWA plasmid[24] using the GatewayTM Technology (Invitrogen). The TCTP gene was amplified by PCR and cloned into a pDONR207 plasmid by BP reaction and then subcloned into pHGGWA plasmid by the LR reaction as described in Busso et al.[24]. The point mutation in TCTP-R21A was introduced by PCR using the following primers 5'-ATCTACAAGATCGCTGAGATCGCG-3' and 5'-

CGCGATCTCAGCGATCTTGTAGAT-3Δ before cloning into a pnEA-TH plasmid for expression of an N-terminal 6His fusion protein using *Nde*I and *BamH*I restriction sites[25]. The cDNA of human Bcl-xL protein, target gene bank number BC019307, was ordered from PlasmID (DANA-FARBER/HARVARD CANCER CENTER). Bcl-xL(Δ27-81ΔCT) (deletion of residues 27-81 and C-terminal 24 residues) was cloned in a pnEA-TH[25] plasmid for expression as a N-terminal 6His fusion protein. Primers used for the deletion of the C-terminal part are 5'-GGATATCCATATGTCTCAGAGCAACCGGG-3' and 5' GAAGATCTCTAGCGTTCCTGGCCC TTTCG-3', including *Nde*I and *Bgl*II restriction sites respectively. Deletion of residues 27 to 81 was obtained by annealing two PCR fragments: one containing the fragment 1-26, and the other the fragment 81-209. Additional primers were used, 5'-CTGCTGCCATCTGACTCCAG-3' and 5'-CTGGAGTCAGATGGCAGCAG-3', as a junction between amino-acids 26 and 81. The point mutant of Bcl-xL(Δ27-81ΔCT)-Y101K was introduced by PCR using the primers 5'-CGACGAATTTGAACTGCGGAAACGGCGGGCATT CAGTGAC-3' and 5'-GTCACTGAAT GCCCGCCGTTTCCGCAGTTCAAATTCGTCG-3'.

Bioluminescence resonance energy transfer (BRET)

BRET assays were performed as indicated in Bah et al.[26]. Briefly, MCF-7 cells seeded on 12-well plates were transfected with 50 ng/well of plasmid pRLuc-TCTP or pRluc-TCTP R21A as indicated and increasing amounts of peYFP-Bcl-xL or peYFP-Bax. Twenty-four hours later, cells were trypsinized and re-seeded onto white 96-well plates, and incubated for 24 hours, prior to treatment with ABT-737 or vehicle alone for an additional 16 hours. Light emissions at 475 nm and 530 nm were measured sequentially using the Mithras fluorescence luminescence detector

LB 940 (Berthold, Thoiry, France) after adding the luciferase substrate coelenterazine H (Uptima, MontLucon, France) at a final concentration of 5 mM. BRET signals (mB.U,milliBRET Unit) were determined by calculating the ratio of the light emitted by the acceptor (530 nm) over the light emitted by the donor (475 nm). Background BRET signals, determined by transient transfection of pCMV-Bcl-xL or pCMV-Bax instead of the corresponding pEYFP plasmids, were subtracted from YFP-specific BRET signals. All data were analyzed using the commercial programme GraphPad PRISM 6.0 (GraphPadSoftware, La Jolla, CA, USA).

Protein expression and purification

Each construct was expressed in *Escherichia coli* strain BL21 (DE3) pRARE2. For protein production, 1L of 2LB (Luria Bertani) medium was inoculated at OD600 of 0.1 by overnight pre-culture at 37°C while cultures were grown until the OD600 reached 0.6-0.8. The soluble fraction of proteins (6His-TCTP-R21A, 6His-Bcl-xL(Δ27-81ΔCT) and 6His-Bcl-xL(Δ27-81ΔCT)-Y101K) was injected onto a TALON His-Tag affinity column (Clontech). Beads were washed with lysis buffer, and targeted proteins were cleaved from the column using thrombin. Bcl-xL was further purified using ion exchange (HiTrap Q-HP, GE Healthcare) and size exclusion (HiLoad Superdex S200, GE Healthcare) chromatography. TCTP-R21A was purified only by size exclusion (HiLoad Superdex S200, GE Healthcare) chromatography. The soluble fraction of GST-TCTP was purified on a glutathione-agarose (Thermo Scientific Pierce) affinity column, washed with lysis buffer containing 2M NaCl and equilibrated with lysis buffer. Intact GST fusion proteins were eluted from the affinity resin using 10mM reduced glutathione then cleaved by TEV protease overnight at 30°C. Cleaved GST was removed by sample loading on a glutathione-agarose (Thermo Scientific Pierce) affinity column while the flowthrough containing TCTP was further purified using ion exchange (HiTrap Q-HP, GE Healthcare) and size exclusion

(HiLoad Superdex S200, GE Healthcare) chromatography. The quality of purified proteins was assessed by SDS-PAGE and Coomassie blue staining. Purified proteins were dialyzed against 10mM Tris-HCl, pH 8, 50mM NaCl, 1mM DTT, flash frozen and stored at -20°C.

Peptides used for *in vitro* assays:

WT-TCTP (11-31): DEMFSDIYKIREIADGLCLEV
R21A-TCTP (11-31): DEMFSDIYKIAEIADGLCLEV
Bax : QPPQDASTKKLSECLKRIGDELDSNMELQRMIADVD
Hybrid Bax/TCTP: QPPQDASTKKLSECIREIADGLCLEV

Peptides used for *in vivo* assays:
WT-TCTP (1-31) : MIIYRDLISHDEMFSDIYKIREIADGLCLEV
TCTP (1-31)-R21A: MIIYRDLISHDEMFSDIYKIAEIADGLCLEV
TCTP (1-20): MIIYRDLISHDEMFSDIYKI
Hybrid Bax-TCTP: MIIYRDLISHDEMKKLSECIREIADGLCLEV

TCTP-FLAG peptides used for *in vivo* assays:
WT-TCTP (1-31)-FLAG : MIIYRDLISHDEMFSDIYKIREIADGLCLEV DYKDDDDK-OH
TCTP (1-31)-R21A-FLAG: MIIYRDLISHDEMFSDIYKIAEIADGLCLEV DYKDDDDK-OH
TCTP-Bax-TCTP-FLAG: MIIYRDLISHDEMKKLSECIREIADGLCLEV DYKDDDDK-OH

The sequence MIIYRDLISH is the one described to allow TCTP penetrate into the cells[23], the *h1* subdomain in the BH3 motif is underlined. Peptides were purchased from Smartox Biotechnology

Complementation and transduction experiments

In order to complement the haploinsufficient TCTP$^{+/-}$ mice with exogenous TCTP, we took advantage of the fact that the first N-terminal fragment of TCTP functions as a Protein Transduction Domain (PTD), enabling TCTP to penetrate into the cell by a mechanism hitherto not well understood[23,27-29]. Thymocytes from wild type or TCTP +/- mice were irradiated (2.5 Gy

of γ irradiation) and cultured, at a concentration of 10^7 cells/ml, overnight in the presence of TCTP protein (Wild type or mutant TCTP protein bearing the mutation R21A) or TCTP peptide (wild type TCTP peptide 1-31, mutant TCTP peptide bearing the mutation R21A, or peptide 1-20) at concentrations ranging from 0-1000nM. For the transduction assays using WT-TCTP-FLAG peptides, R21-mutant TCTP-FLAG peptides and TCTP/Bax-hybrid-FLAG peptides, the concentrations were 10 microM, 50 microM and 100 microM.

Immunohistochemistry

γ-irradiated thymocytes were fixed by adding 500ul of PFA 4% and incubated for 20 min at room temperature. The cells were washed twice with PBS and permeabilized for 10 min with 0.2% triton X-100 in PBS at room temp. The cells were washed twice with PBS. Proteins were blocked in Blocking buffer (1% BSA 0.5% Tween in PBS) 1h at room temperature. The primary antibody was added with blocking buffer and placed in a rotating wheel overnight in a cold room.

Cells were then washed 3 times with PBS and incubated with secondary antibody with blocking buffer 1h at room temperature. The cells were washed 3 times with PBS and suspended in 200 µL PBS and used for Flow Cytometry or further used for immunohistochemistry: 5 µl of each sample were mixed with 5 µl of ProLong® Gold antifade reagent (Life Technologies) and visualized, using the *IX73* inverted fluorescence microscope and the cellSens™ Dimension software (Olympus).

Flow Cytometry

FACS analysis (BD Accuri™ C6 Cytometer (BD Biosciences) of incorporation of exogenous

recombinant TCTP protein into γ-irradiated thymocytes. Polyclonal rabbit anti-TCTP antibodies (TCTP1)[30] are use de detect TCTP and anti-rabbit-FITC was used as secondary antibody (Abcam).

To detect the WT-TCTP-FLAG peptides, R21-mutant TCTP-FLAG peptides and TCTP/Bax-hybrid-FLAG peptides, rabbit anti-FLAG (Sigma) and anti-rabbit-FITC (Abcam) were used. Since the combination of these antibody generated a significant background (Supplementary Figure 14D), we used as an alternative using anti-FLAG antibodies directly labeled with-phyco-*erythrin* (Abcam) that generated no significant background (Supplementary Figure 14F).

Isothermal Titration Calorimetry experiments

Titrations were done by isothermal titration calorimetry (ITC) assays using an ITC200 calorimeter from GE-Microcal. Bcl-xL was used at 0.065 mM in 50mM bicarbonate ammonium pH 9. TCTP11-31 was used at 1 mM in the same buffer. Titrations were carried out at 30°C. Data were analyzed using Microcal Origin software provided by the ITC manufacturer (GE-Microcal). The same experimental design was used to measure Bax/Bcl-xL and Bax-TCTP hybrid/Bcl-xL binding.

Crystallization

High-throughput crystallization screening was performed using a Mosquito liquid transfer robot (TTP Labtech) and the sitting-drop vapor diffusion method. Crystals were obtained at 20°C by mixing 0.2μL of reservoir solution (100mM Pipes pH 7, 1.5M Tris-sodium citrate) with 0.2μL of 300 μM of Bcl-xL (Δ27-81ΔCT) + 1mM TCTP11-31 solution in buffer containing 50mM ammonium bicarbonate pH 9.3 and by equilibrating the mixture against 40μl of reservoir solution.

X-ray data collection, structure determination, and refinement

Crystals were cryoprotected with a 15% Polyethylene glycol 1.2M tris-sodium citrate 100mM Pipes pH 7 solution and flash-frozen in liquid nitrogen. A 2.1Å resolution data set was collected at 100 K on beamline ID29 at the European Synchrotron Radiation Facility with a Pilatus 6MF detector. The crystal belongs to space group $P4_12_12$ with unit cell dimensions a=100.36Å, b=100.36Å, c=105.04Å and α=β=γ=90°. Diffraction data were processed, integrated, and scaled with XDS[31] and HKL2000[32]. The structure of human Bcl-xL/$TCTP_{11-31}$ complex was solved by molecular replacement using the program Phenix[33] and coordinates from the human Bcl-xL-Beclin complex (Protein Data Bank code 2P1L[14]) as a search model. The model was built with Coot[34] and refined with Phenix[33] and Buster[35]. The statistics are summarized in **Table 1** of the main text. Model geometry and validation were done using MolProbity[36]. Ramachandran plot analysis showed that 98% of all residues were in the most favoured and the other 2% in additionally allowed conformations. All other crystallographic calculations were carried out with the CCP4 package[37]. Structural figures were prepared with PyMOL (Delano Scientific, http://www.pymol.org). Coordinates of the refined structural model and structure factors have been deposited to the Protein Data Bank (PDB) with the accession code 4Z9V.

Interaction experiments: When not specified, proteins tested for binding experiments were mixed at 100µM, dialyzed against 10mM CHES pH 9, 50mM NaCl, 1mM DTT or TCEP, depending on the analysis, and heated overnight at 30°C.

Gel filtration. For binding experiments, 100 to 200PL of protein mixture were loaded on a size exclusion chromatography (S200, GE Healthcare) column, while elution profiles were measured by UV absorption at 280nm.

Size-Exclusion Chromatography coupled to Multi-Angle Light Scattering (SEC-MALS)

SEC-MALS experiments were performed on a multi-angle light scattering detector (miniDAWN TREOS, Wyatt Technologies) coupled in-line with SEC and an interferometric refractometer (Optilab T-rEX, Wyatt Technologies). A Superdex S200 10/300 GL column (total volume 24 mL, GE Healthcare) with a flow rate of 0.5 mL/min was used to separate the sample before performing MALS measurements. Experiments were done with 90µL of protein sample at a concentration of 100µM in 10mM CHES pH 9, 50mM NaCl or 250µM in 10mM Tris pH 8, 25 mM NaCl for Bcl-xL(Δ27-81ΔCT)+TCTP, respectively.

For MALS experiments, the molar mass was determined by construction of Debye plot using Zimm formalism (plot of K*c/R(T) as a function of sin2(T/2)) at 0.5 and 1sec data intervals, for Superdex S200 column. Data analysis was performed using the ASTRA 5.3.4 software (Wyatt Technologies).

Native mass spectrometry. Prior to any mass spectrometry experiment, protein buffer was exchanged twice against a 50mM ammonium acetate (NH4Ac) solution at pH 9.0 using microcentrifuge gel filtration columns (Zeba 0.5ml, Thermo Scientific, Rockford, IL). Protein concentration was determined spectrophotometrically. NanoESI-MS measurements were carried out on an electrospray quadrupole-time-of-flight mass spectrometer (Synapt G2 HDMS, Waters, Manchester, UK) equipped with an automated chip-based nanoESI source (Triversa Nanomate, Advion Biosciences, Ithaca, NY) operating in the positive ion mode. External calibration was performed with the multiple charged ions produced by a 2µM horse heart myoglobin solution diluted in a 1:1 (v/v) water: acetonitrile mixture acidified with 1% (v/v) formic acid. Purity and

homogeneity of samples were first assayed in denaturing conditions by diluting the proteins to 2µM in a 1:1 (v/v) water/acetonitrile mixture acidified with 1% (v/v) formic acid (**Supplementary Table 1**). Analyses in native conditions were then performed by diluting proteins to 5 µM in NH4Ac buffer 50mM at pH 9.0 (adjusted with ammonia). Experiments were realized after careful optimization of instrumental parameters. Particularly, the pressure in the first pumping stage was raised up to 6 mbar using a throttling valve and the acceleration voltage applied on the sample cone was set to 40 V. Data analysis were performed with MassLynx 3.5 (Waters, Manchester, UK).

***in vitro* system**: purified recombinant proteins are used to permeabilize liposomes as described before[38,39].

Cytochrome *c* release assay. Mitochondria from mouse liver were purified as described by Eskes et al[40]. The mitochondria (50 µg protein) were incubated for 15 min at 30°C in 100µL KCl buffer (125mM KCl, 0.5mM EGTA, 10mM HEPES-KOH [pH7.4], 4mM $MgCl_2$) supplemented with 5nM recombinant caspase-8-cleaved Bid (tBid), 50nM Bax, 300nM BclxL and in the presence or absence of TCTP protein or peptides. The reaction mixtures were then centrifuged at 10,000 *g* for 10min at 4°C to collect the mitochondria. Mitochondrial pellets and the corresponding supernatant fractions were subjected to SDS-polyacrylamide gel electrophoresis and analyzed by Western blotting for cytochrome c.

Supplementary References

1. Susini, L. *et al.* TCTP protects from apoptotic cell death by antagonizing bax function. *Cell Death Differ.* **15**, 1211-1220 (2008).
2. O'Neill, J. W., Manion, M. K., Maguire, B. & Hockenbery, D. M. BCL-XL dimerization by three-dimensional domain swapping. *J. Mol. Biol.* **356**, 367-381 (2006).
3. Denisov, A. Y., Sprules, T., Fraser, J., Kozlov, G. & Gehring, K. Heat-induced dimerization of BCL-xL through alpha-helix swapping. *Biochemistry* **46**, 734-740 (2007).
4. Kvansakul, M. *et al.* Vaccinia virus anti-apoptotic F1L is a novel Bcl-2-like domain-swapped dimer that binds a highly selective subset of BH3-containing death ligands. *Cell Death Differ.* **15**, 1564-1571 (2008).
5. Schrodinger, LLC. *The PyMOL Molecular Graphics System, Version 1.3r1* (2010).
6. Minn, A. J. *et al.* Bcl-xL regulates apoptosis by heterodimerization-dependent and -independent mechanisms. *EMBO J.* **18**, 632-643 (1999).
7. Yang, H., Filipovic, Z., Brown, D., Breit, S. N. & Vassilev, L. T. Macrophage inhibitory cytokine-1: a novel biomarker for p53 pathway activation. *Mol. Cancer Ther.* **2**, 1023-1029 (2003).
8. Zhang, D., Li, F., Weidner, D., Mnjoyan, Z. H. & Fujise, K. Physical and functional interaction between myeloid cell leukemia 1 protein (MCL1) and Fortilin. The potential role of MCL1 as a fortilin chaperone. *J. Biol. Chem.* **277**, 37430-37438 (2002).
9. Yang, Y. *et al.* An N-terminal region of translationally controlled tumor protein is required for its antiapoptotic activity. *Oncogene* **24**, 4778-4788 (2005).
10. Lee, E. F. *et al.* Crystal structure of ABT-737 complexed with Bcl-xL: implications for selectivity of antagonists of the Bcl-2 family. *Cell Death Differ.* **14**, 1711-1713 (2007).
11. Zhai, D., Jin, C., Satterthwait, A. C. & Reed, J. C. Comparison of chemical inhibitors of antiapoptotic Bcl-2-family proteins. *Cell Death Differ.* **13**, 1419-1421 (2006).
12. Aouacheria, A., Brunet, F. & Gouy, M. Phylogenomics of life-or-death switches in multicellular animals: Bcl-2, BH3-Only, and BNip families of apoptotic regulators. *Mol. Biol. Evol.* **22**, 2395-2416 (2005).
13. Ambrosi, E. *et al.* Structural changes in the BH3 domain of SOUL protein upon interaction with the anti-apoptotic protein Bcl-xL. *Biochem. J.* **438**, 291-301 (2011).

14. Oberstein, A., Jeffrey, P. D. & Shi, Y. Crystal structure of the Bcl-XL-Beclin 1 peptide complex: Beclin 1 is a novel BH3-only protein. *J. Biol. Chem.* **282**, 13123-13132 (2007).

15. Okamoto, T. *et al.* Stabilizing the pro-apoptotic BimBH3 helix (BimSAHB) does not necessarily enhance affinity or biological activity. *ACS Chem. Biol.* **8**, 297-302 (2013).

16. Sattler, M. *et al.* Structure of Bcl-xL-Bak peptide complex: recognition between regulators of apoptosis. *Science* **275**, 983-986 (1997).

17. Petros, A. M. *et al.* Rationale for Bcl-xL/Bad peptide complex formation from structure, mutagenesis, and biophysical studies. *Protein Sci.* **9**, 2528-2534 (2000).

18. Follis, A. V. *et al.* PUMA binding induces partial unfolding within BCL-xL to disrupt p53 binding and promote apoptosis. *Nat. Chem. Biol.* **9**, 163-168 (2013).

19. Feng, W., Huang, S., Wu, H. & Zhang, M. Molecular basis of Bcl-xL's target recognition versatility revealed by the structure of Bcl-xL in complex with the BH3 domain of Beclin-1. *J. Mol. Biol.* **372**, 223-235 (2007).

20. Moroy, G., Martin, E., Dejaegere, A. & Stote, R. H. Molecular basis for Bcl-2 homology 3 domain recognition in the Bcl-2 protein family: identification of conserved hot spot interactions. *J. Biol. Chem.* **284**, 17499-17511 (2009).

21. Fahling, M., Perlewitz, A., Doller, A. & Thiele, B. J. Regulation of collagen prolyl 4-hydroxylase and matrix metalloproteinases in fibrosarcoma cells by hypoxia. *Comp Biochem Physiol C Toxicol Pharmacol* **139**, 119-126 (2004).

22. Montessuit, S. *et al.* Membrane remodeling induced by the dynamin-related protein Drp1 stimulates Bax oligomerization. *Cell* **142**, 889-901 (2010).

23. Kim, H. Y. *et al.* The cell penetrating ability of the proapoptotic peptide, KLAKLAKKLAKLAK fused to the N-terminal protein transduction domain of translationally controlled tumor protein, MIIYRDLISH. *Biomaterials* **32**, 5262-5268 (2011).

24. Busso, D., Delagoutte-Busso, B. & Moras, D. Construction of a set Gateway-based destination vectors for high-throughput cloning and expression screening in Escherichia coli. *Anal Biochem* **343**, 313-321 (2005).

25. Diebold, M. L., Fribourg, S., Koch, M., Metzger, T. & Romier, C. Deciphering correct strategies for multiprotein complex assembly by co-expression: application to complexes as large as the histone octamer. *J Struct Biol* **175**, 178-188 (2011).

26. Bah, N. *et al.* Bcl-xL controls a switch between cell death modes during mitotic arrest. *Cell Death Dis* **5**, e1291 (2014).

27. Maeng, J., Kim, H. Y., Shin, D. H. & Lee, K. Transduction of translationally controlled tumor protein employing TCTP-derived protein transduction domain. *Anal Biochem* **435**, 47-53 (2013).

28. Jenkins, E. O., Schiff, D., Mackman, N. & Key, N. S. Venous thromboembolism in malignant gliomas. *J Thromb Haemost* **8**, 221-227 (2010).

29. Kim, M. *et al.* A protein transduction domain located at the NH2-terminus of human translationally controlled tumor protein for delivery of active molecules to cells. *Biomaterials* **32**, 222-230 (2011).

30. Amson, R. *et al.* Reciprocal repression between P53 and TCTP. *Nat Med* **18**, 91-99 (2012).

31. Kabsch, W. XDS. *Acta Crystallogr. Sect. D Biol. Crystallogr.* **66**, 125-132 (2010).

32. Otwinowski, Z. & Minor, W. in *Macromolecular Crystallography.* Vol. 276A *Methods in Enzymology* (eds C.W. Carter & R.M. Sweet) 307-326 (Academic Press, 1997).

33. Adams, P. D. *et al.* PHENIX: a comprehensive Python-based system for macromolecular structure solution. *Acta Crystallogr. Sect. D Biol. Crystallogr.* **66**, 213-221 (2010).

34. Emsley, P. & Cowtan, K. Coot: model-building tools for molecular graphics. *Acta Crystallogr. Sect. D Biol. Crystallogr.* **60**, 2126-2132 (2004).

35. BUSTER version 2.10.0 (Global Phasing Ltd., Cambridge, United Kingdom, 2011).

36. Chen, V. B. *et al.* MolProbity: all-atom structure validation for macromolecular crystallography. *Acta Crystallogr D Biol Crystallogr* **66**, 12-21 (2010).

37. Collaborative Computational Project, N. The CCP4 suite: programs for protein crystallography. *Acta Crystallogr. Sect. D Biol. Crystallogr.* **50**, 760-763 (1994).

38. Aranovich, A. *et al.* Differences in the mechanisms of proapoptotic BH3 proteins binding to Bcl-XL and Bcl-2 quantified in live MCF-7 cells. *Mol. Cell* **45**, 754-763 (2012).

39. Billen, L. P., Kokoski, C. L., Lovell, J. F., Leber, B. & Andrews, D. W. Bcl-XL inhibits membrane permeabilization by competing with Bax. *PLoS Biol* **6**, e147 (2008).

40. Eskes, R., Desagher, S., Antonsson, B. & Martinou, J. C. Bid induces the oligomerization and insertion of Bax into the outer mitochondrial membrane. *Mol. Cell. Biol.* **20**, 929-935 (2000).
